# Supplementary material for: Multiplexed detection of viral antigen and RNA using nanopore sensing and encoded molecular probes
Source: Nat Commun. 2023 Nov 14;14:7362. doi: 10.1038/s41467-023-43004-9 (PMC10646045; doi:10.1038/s41467-023-43004-9)
Supplement: Supplementary file 1 — Supplementary Information [file 41467_2023_43004_MOESM1_ESM.pdf]

Supplementary Information

Multiplexed detection of viral antigen and RNA using nanopore sensing and encoded molecular probes

Ren Ren[1,2,#], Shenglin Cai[1,3,#,*], Xiaona Fang[4], Xiaoyi Wang[1], Zheng Zhang[4], Micol Damiani[1], Charlotte Hudlerova[1], Annachiara Rosa[5,12], Joshua Hope[5], Nicola J Cook[5], Peter Gorelkin[6], Alexander Erofeev[6], Pavel Novak[7], Anjna Badhan[8], Michael Crone[9], Paul Freemont[9], Graham Taylor[8], Longhua Tang[10], Christopher Edwards[2,7], Andrew Shevchuk[2], Peter Cherepanov[5,8], Zhaofeng Luo[4], Weihong Tan[4,*], Yuri Korchev[2,11], Aleksandar P. Ivanov[1,*], Joshua B. Edel[1,*]

[1]Department of Chemistry, Imperial College London, Molecular Science Research Hub, White City Campus, 82 Wood Lane, London W12 0BZ, UK.
[2]Department of Metabolism, Digestion and Reproduction, Imperial College London, Hammersmith Campus, Du Cane Road, London W12 0NN, UK.
[3]Yusuf Hamied Department of Chemistry, University of Cambridge, Lensfield Road, Cambridge CB2 1EW, UK.
[4]The Key Laboratory of Zhejiang Province for Aptamers and Theranostics, Aptamer Selection Center, Hangzhou Institute of Medicine (HIM), Chinese Academy of Sciences, Hangzhou, Zhejiang 310022, China
[5]The Chromatin Structure and Mobile DNA Laboratory, The Francis Crick Institute, London, UK
[6]National University of Science and Technology "MISIS", Leninskiy Prospect 4, 119991, Moscow, Russian Federation
[7]ICAPPIC Limited, The Fisheries, Mentmore Terrace, London, UK, E8 3PN.
[8]Molecular Diagnostic Unit, Section of Virology, Department of Infectious Disease, Faculty of Medicine, Imperial College London.
[9]Section of Structural and Synthetic Biology, Department of Infectious Disease, Faculty of Medicine, Imperial College London, UK.
[10]State Key Laboratory of Modern Optical Instrumentation, College of Optical Science and Engineering, International Research Center for Advanced Photonics, Zhejiang University, Hangzhou 310027, China.
[11]Nano Life Science Institute (WPI-NanoLSI), Kanazawa University, Kakuma-machi, Kanazawa, 920-1192, Japan
[12]Wolfson Education Centre, Faculty of Medicine, Imperial College London, London W12 0NN, UK.

*Correspondence: joshua.edel@imperial.ac.uk; alex.ivanov@imperial.ac.uk; tan@hnu.edu.cn; shenglin.cai15@imperial.ac.uk
#These authors contributed equally: Ren Ren, Shenglin Cai

Supplementary Figures

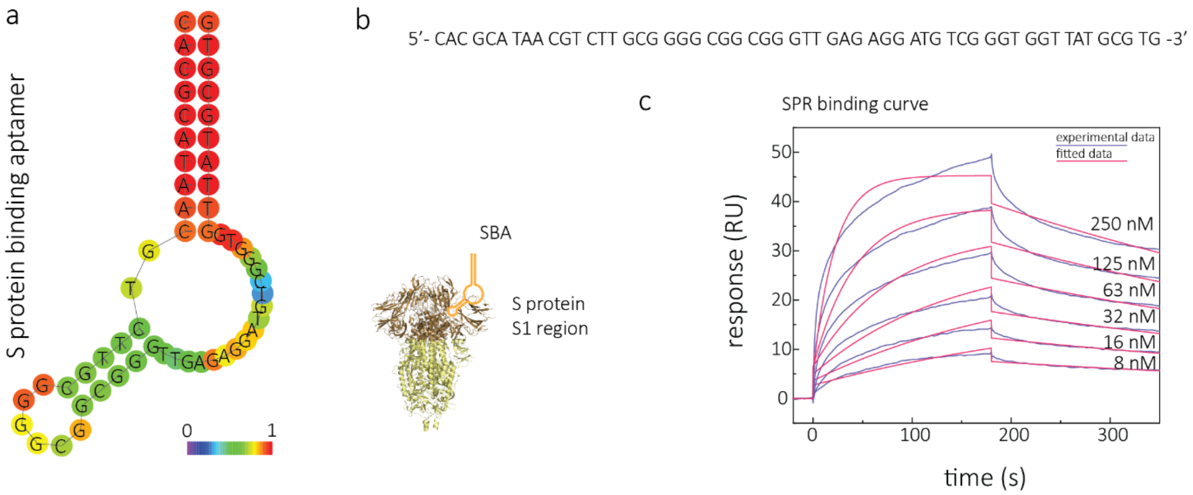

Supplementary Fig. 1 | SBA aptamer and its binding to S protein of SARS-CoV-2.

(a) Graphic representation showing the predicted structure of SBA aptamer using Centroid Secondary Structure. The colour bar represents the predicted probability using the lowest free energy. The graphical representation of the S protein and its binding to SBA are shown on the right side. **(b)** Sequence of SBA. **(c)** SPR response curves of SBA upon adding S protein with concentrations of 8, 16, 32, 63, 125, and 250 nM, respectively. Blue lines are experimental measurement data, and red lines are fitted data. The K_D for this SBA binding to the S1 domain was calculated to be 9.86 nM. Source data is provided as a Source Data file.

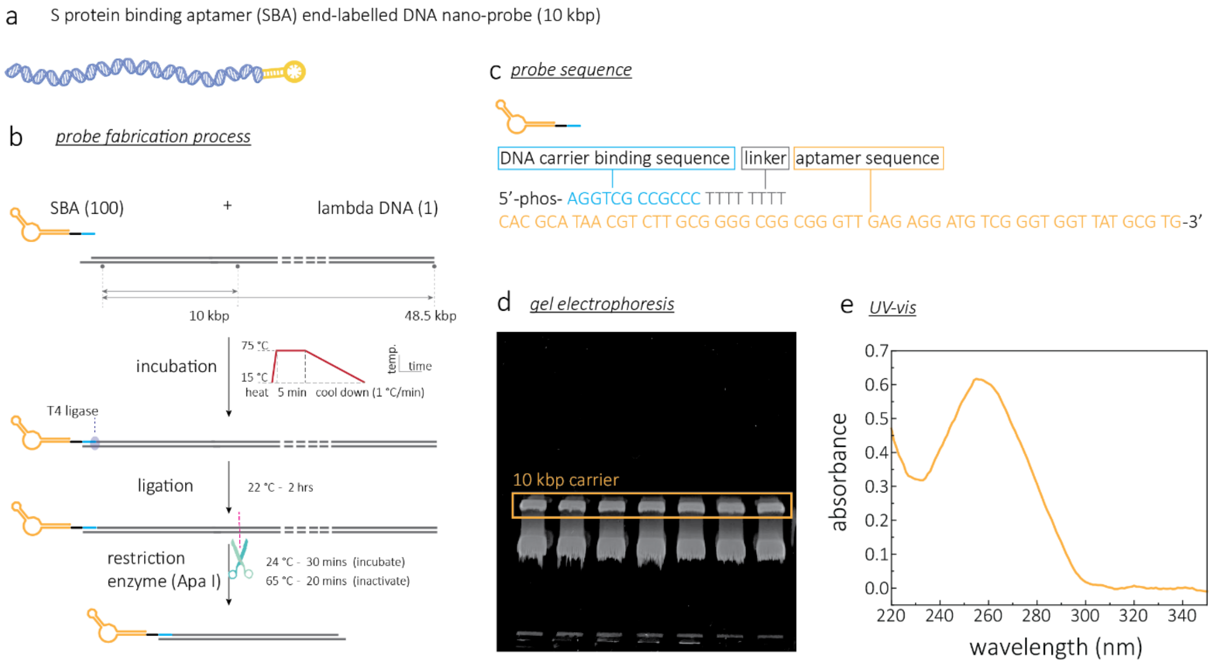

Supplementary Fig. 2 | Preparation and characterisation of SBA end-labelled DNA molecular probe.

(a) Schematic showing the 10 kbp DNA molecular probe end-labelled with SBA. **(b)** Schematic illustration of the preparation process of the SBA-labelled molecular probe from λ-DNA. **(c)** Schematic and sequence of the SBA probe used for attaching to the 10 kbp DNA strand. The probe consists of three parts: the DNA carrier binding sequence (sky blue), the 8-T linker (grey), and the aptamer sequence (yellow). **(d)** Gel electrophoresis showing the separation of the 10 kbp DNA molecular probe from the 38.5 kbp residues. **(e)** UV-Vis spectrum for the molecular probe extracted from gel electrophoresis. The concentration of the molecular probe was estimated using the absorbance at 260 nm. Source data are provided as a Source Data file.

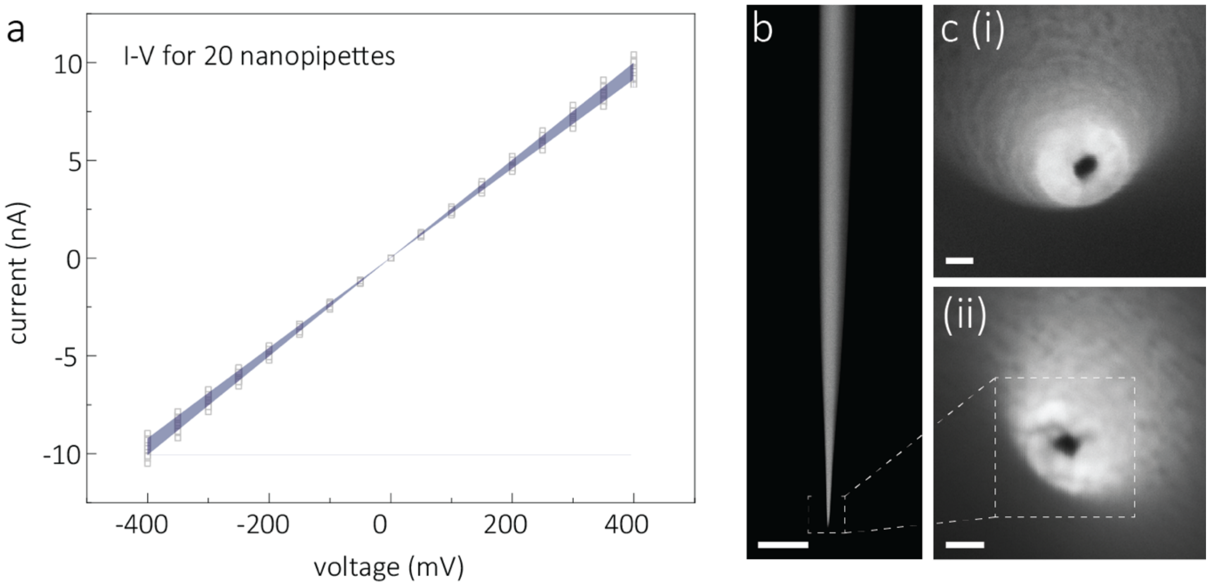

Supplementary Fig. 3 | Conductance and SEM characterisation of nanopipettes.

(a) I-V characteristics of nanopores were performed before each experiment in 1 M KCl. The conductance of the nanopore was calculated from the slope by linear fitting of the I-V curves as G = 15.4 ± 1.2 nS (n = 20). The grey-shaded region indicates one standard deviation (n = 20). **(b)** SEM image of a side view of a typical nanopipette showing a conical shape and tapered tip. (scale bar: 10 µm) **(c)** Close-up SEM image showing the cross-section of nanopipette (scale bar: (i) 20 nm, (ii) 20 nm). The diameter of nanopipettes measured by SEM is 15 ± 3 nm (n = 5). Source data are provided as a Source Data file.

I-V characterisation was performed to approximate the pore size and determine pore conductance prior to translocation experiments. The equation below was used to estimate the pore size.[1]

$$R_{\mathrm{p}} = \frac{1}{\kappa \pi r_{\mathrm{i}} \tan\theta} + R_{\mathrm{access}} \approx \frac{1}{\kappa \pi r_{\mathrm{i}} \tan\theta} + \frac{1}{4\kappa r_{\mathrm{i}}}$$

where R_{p} is the nanopipette resistance, r_{i} is the inner pipette radius, κ is the solution conductivity, and θ is the inner nanopipette half-cone angle.

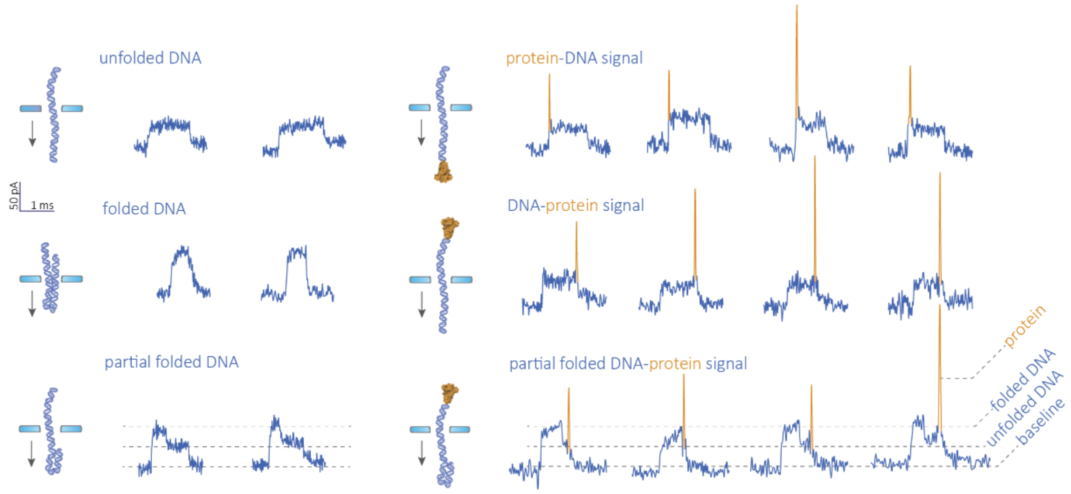

Supplementary Fig. 4 | Example translocation events for SBA-modified molecular probes without and with S protein.

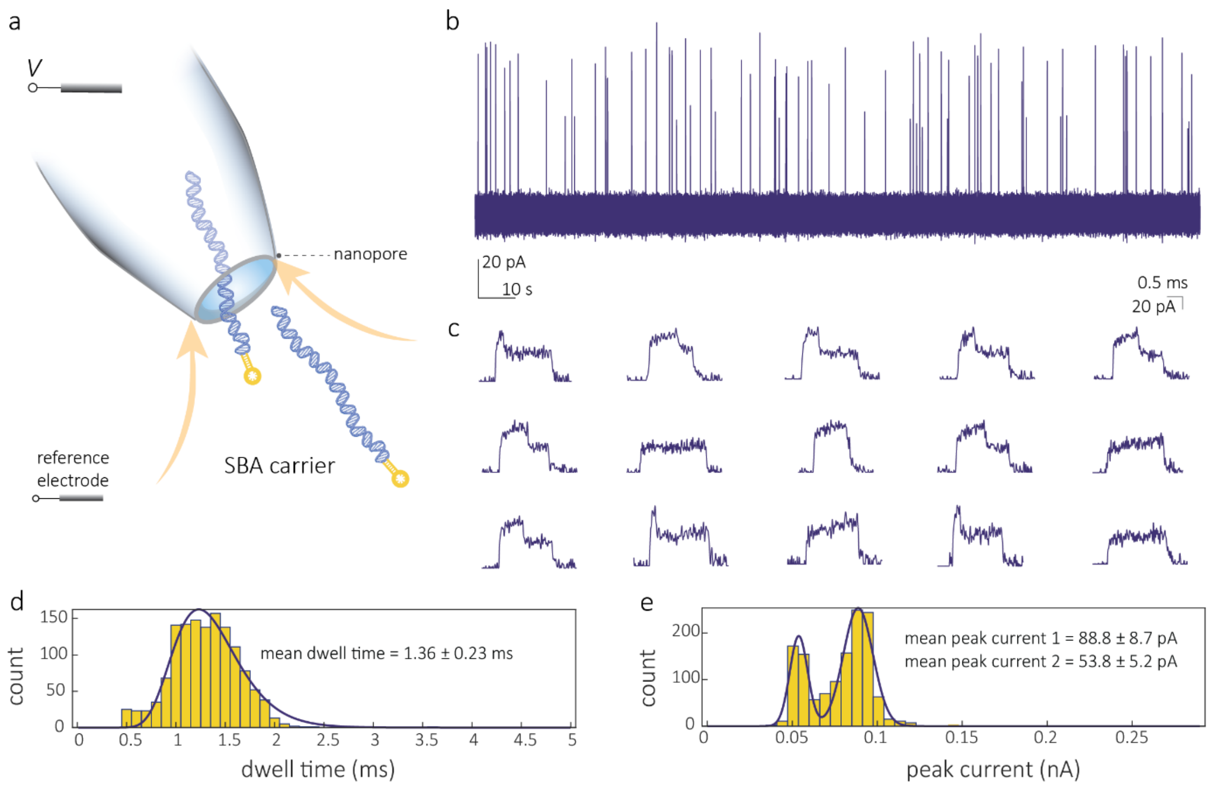

Supplementary Fig. 5 | Translocation of SBA-labelled DNA molecular probes only.

Schematic **(a)** and representative current-time trace **(b)** showing the SBA-labelled molecular probe (10 kbp) translocating through a nanopore in the absence of target protein. **(c)** Zoom-in view of typical translocation events. **(d)** Histograms of dwell time for the translocation of SBA-labelled molecular probes. **(e)** Histograms of peak amplitude for the translocation events. Two distinct peaks were observed, with one at 53.8 ± 5.2 pA (linear DNA) and the other at 88.8 ± 8.7 pA (folded DNA). All the translocation experiments were performed using 200 pM DNA in 2 M LiCl buffer (5 mM $MgCl_2$, 10 mM Tris-HCl, 1 mM EDTA, pH = 8) at an applied potential bias of 300 mV.

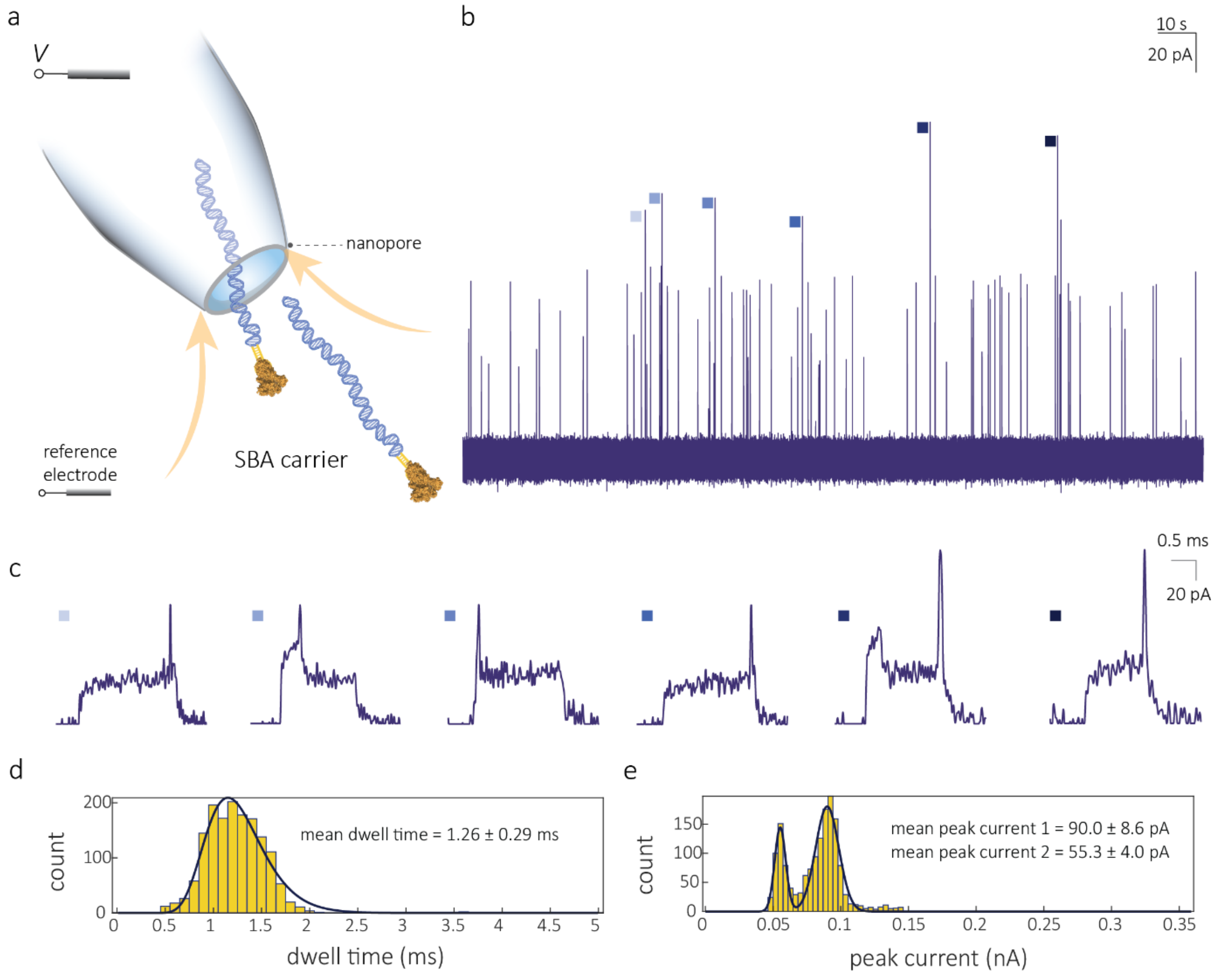

Supplementary Fig. 6 | Translocation of SBA-labelled DNA molecular probe bound to S protein.

Schematic **(a)** and representative current-time trace **(b)** showing the SBA-labelled molecular probe (200 pM) translocating through a nanopore in the presence of S protein (20 nM). **(c)** Zoom-in view of typical translocation events for S protein bound to SBA-labelled molecular probe marked in **(b)**. **(d)** Histograms of dwell time. **(e)** Histograms of peak amplitude for the translocation events. Two peaks were observed at 55.3 ± 4.0 pA and 90.0 ± 8.6 pA, respectively. All the translocation experiments were performed using 200 pM DNA in 2 M LiCl buffer (5 mM $MgCl_2$, 10 mM Tris-HCl, 1 mM EDTA, pH = 8) at an applied potential bias of 300 mV.

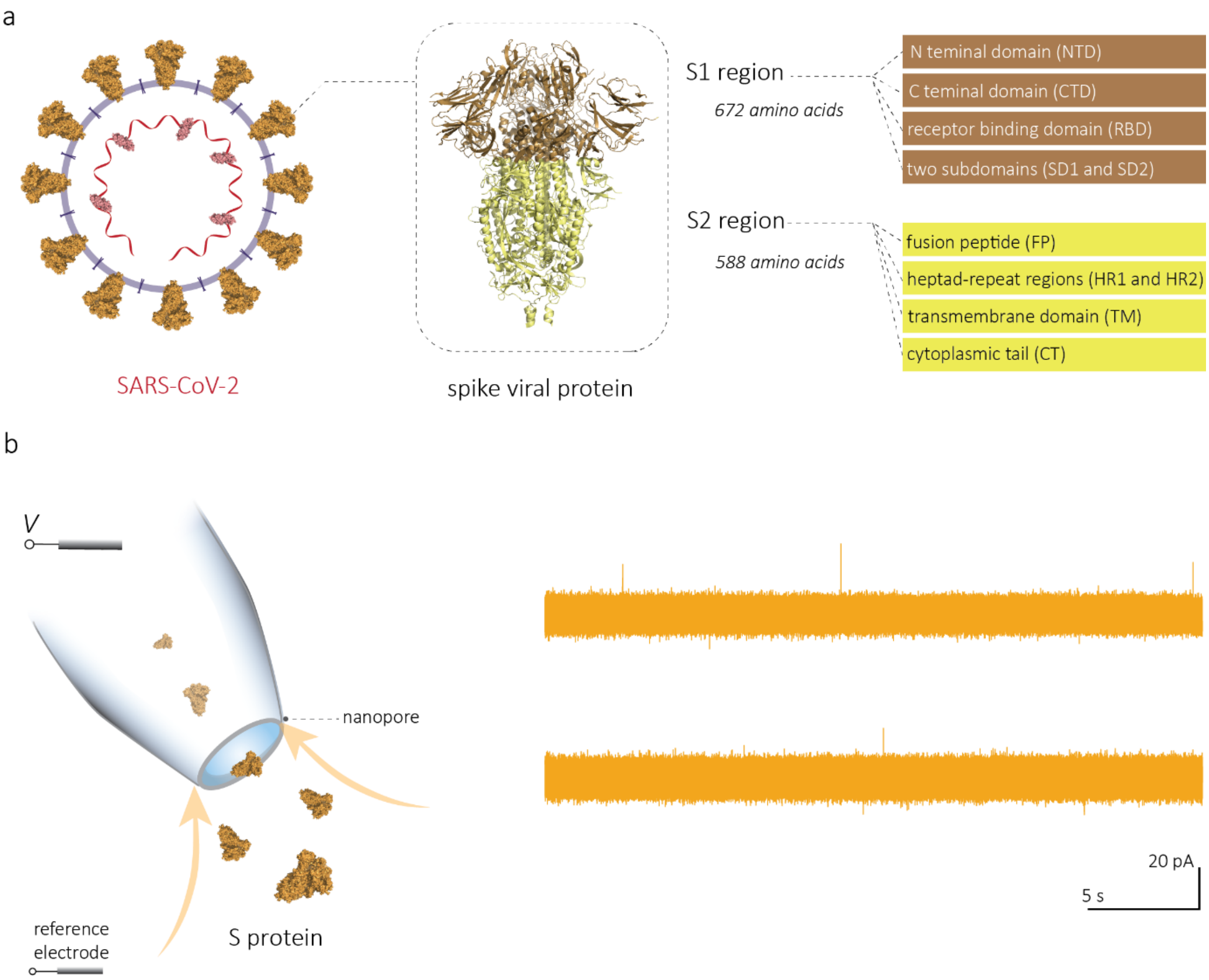

Supplementary Fig. 7 | Model of the S protein and current time trace.

(a) Schematic representation of SARS-CoV-2 along with the spike protein. The whole S protein size was determined by CryoEM to be approximately 16 nm.[2] The S1 subunit was used in this manuscript and is approximately 8-10 nm (S1/S=672/1260 amino acids). **(b)** Schematic for the translocation of S protein only (20 nM). Representative current-time traces for the S protein translocations are shown on the right side, indicating that no translocation events could be detected. Translocation experiments were performed with 20 nM S protein in 2 M LiCl buffer (5 mM $MgCl_2$, 10 mM Tris-HCl, 1 mM EDTA, pH = 8) at an applied potential bias of 300 mV.

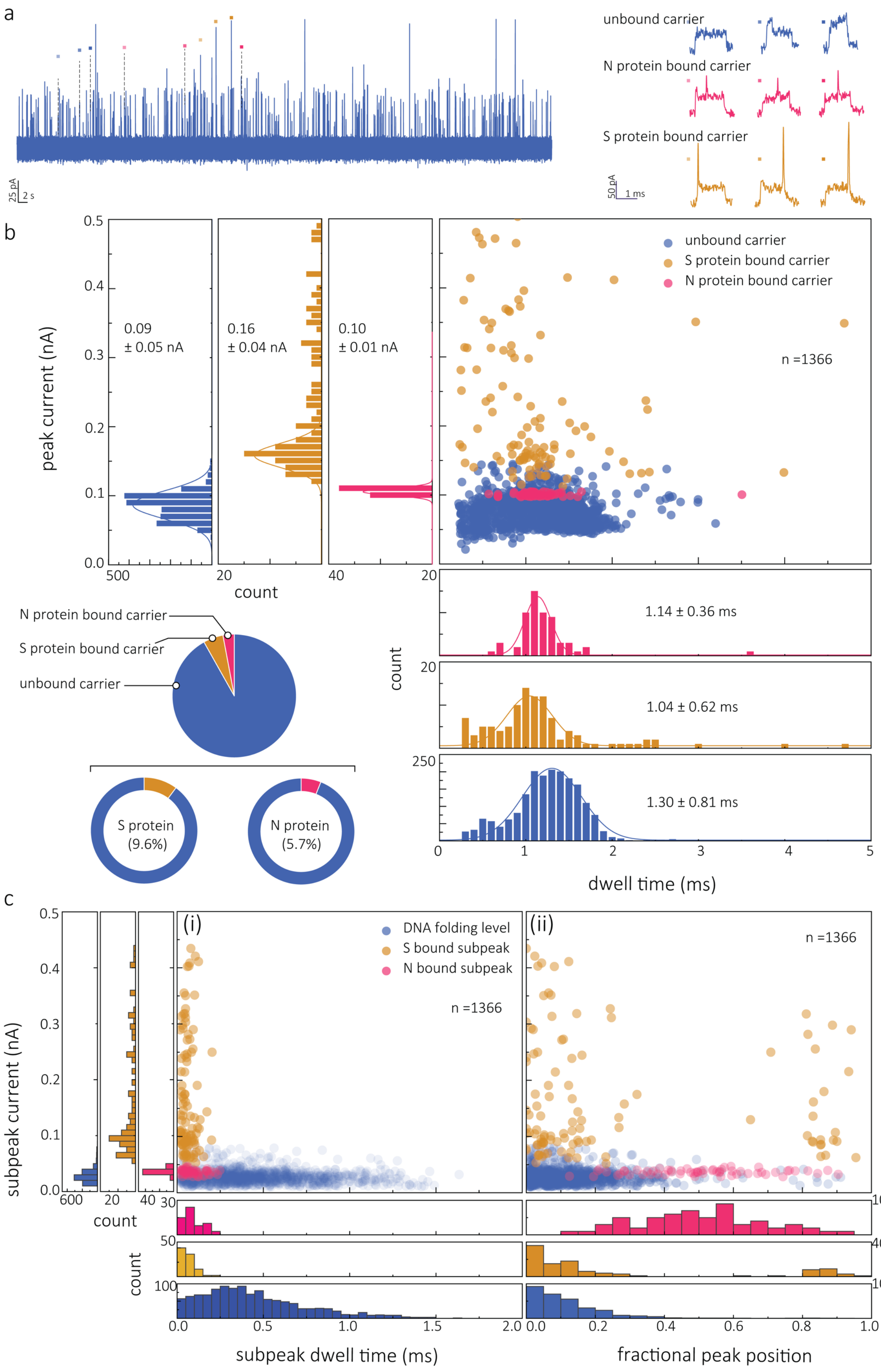

Supplementary Fig. 8 | Subpeak analysis of S, N protein, and folded DNA

(a) Representative trace and individual events for the translocation of the unbound probe (blue), S protein-bound probe (yellow), and N protein-bound probe (red). **(b)** Scatter plots and histograms are shown for the total peak current and dwell time. (c) Scatter plots and histograms are shown for the subpeak amplitude (amplitude difference between the peak amplitude and DNA level), subpeak dwell time, and fractional position of the subpeak. As discussed in the main text, these 3 variables were used to discriminate between folding events and subpeaks associated with the S and N proteins. The translocation experiments were performed using molecular probes at a concentration of 200 pM in 2 M LiCl buffer (5 mM $MgCl_2$, 10 mM Tris-HCl, 1 mM EDTA, pH = 8) at an applied potential bias of 300 mV.

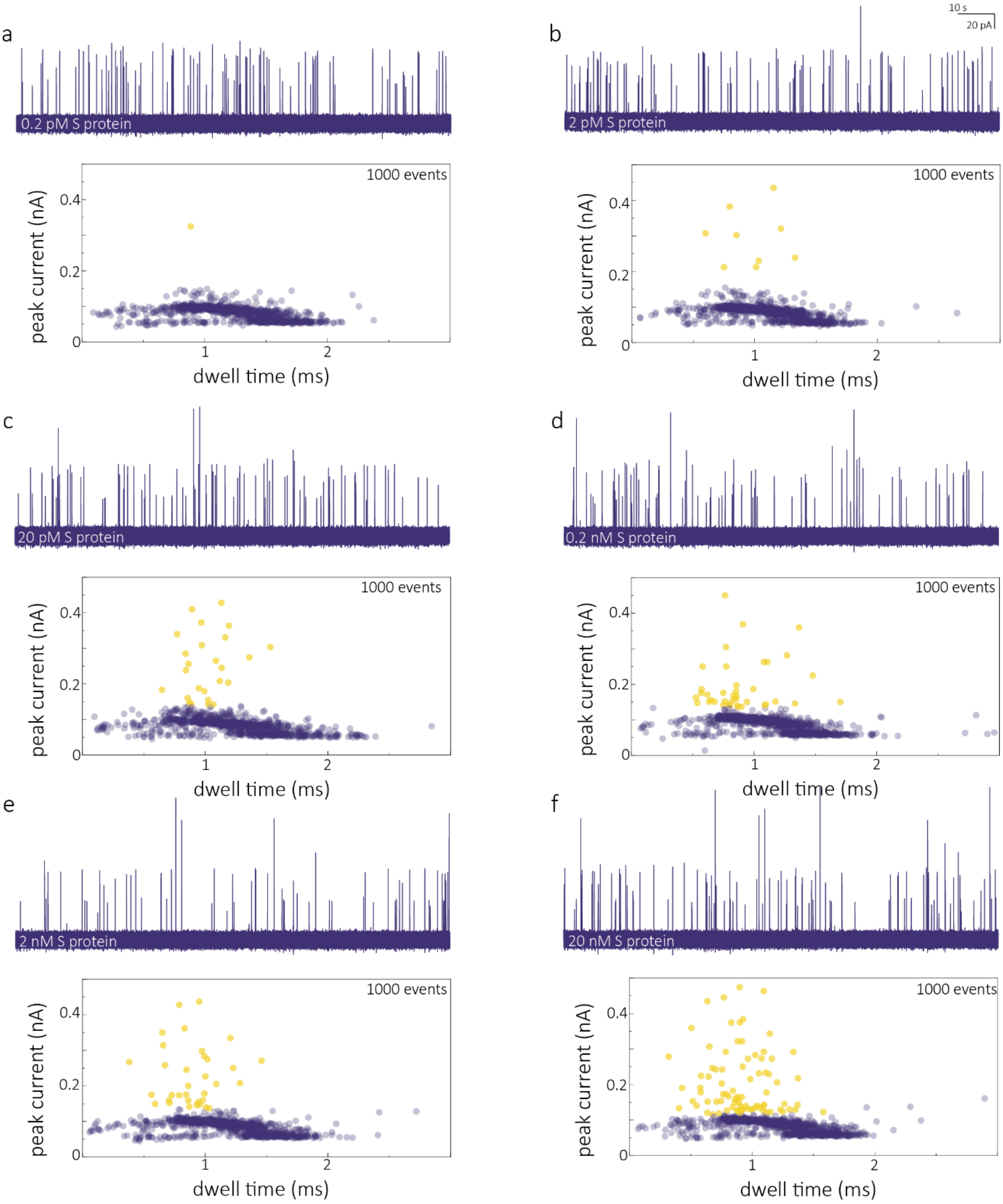

Supplementary Fig. 9 | Concentration dependence of S protein.

Representative current-time traces for SBA-labelled molecular probe translocations in the presence of **(a)** 0.2 pM, **(b)** 2 pM, **(c)** 20 pM, **(d)** 0.2 nM, **(e)** 2 nM, and **(f)** 20 nM of S protein. The scatter plots of peak current versus dwell time for 1000 events are shown below, with protein-bound events highlighted in yellow. All the translocation experiments were performed using molecular probes at a concentration of 200 pM in 2 M LiCl buffer (5 mM $MgCl_2$, 10 mM Tris-HCl, 1 mM EDTA, pH = 8) at an applied potential bias of 300 mV.

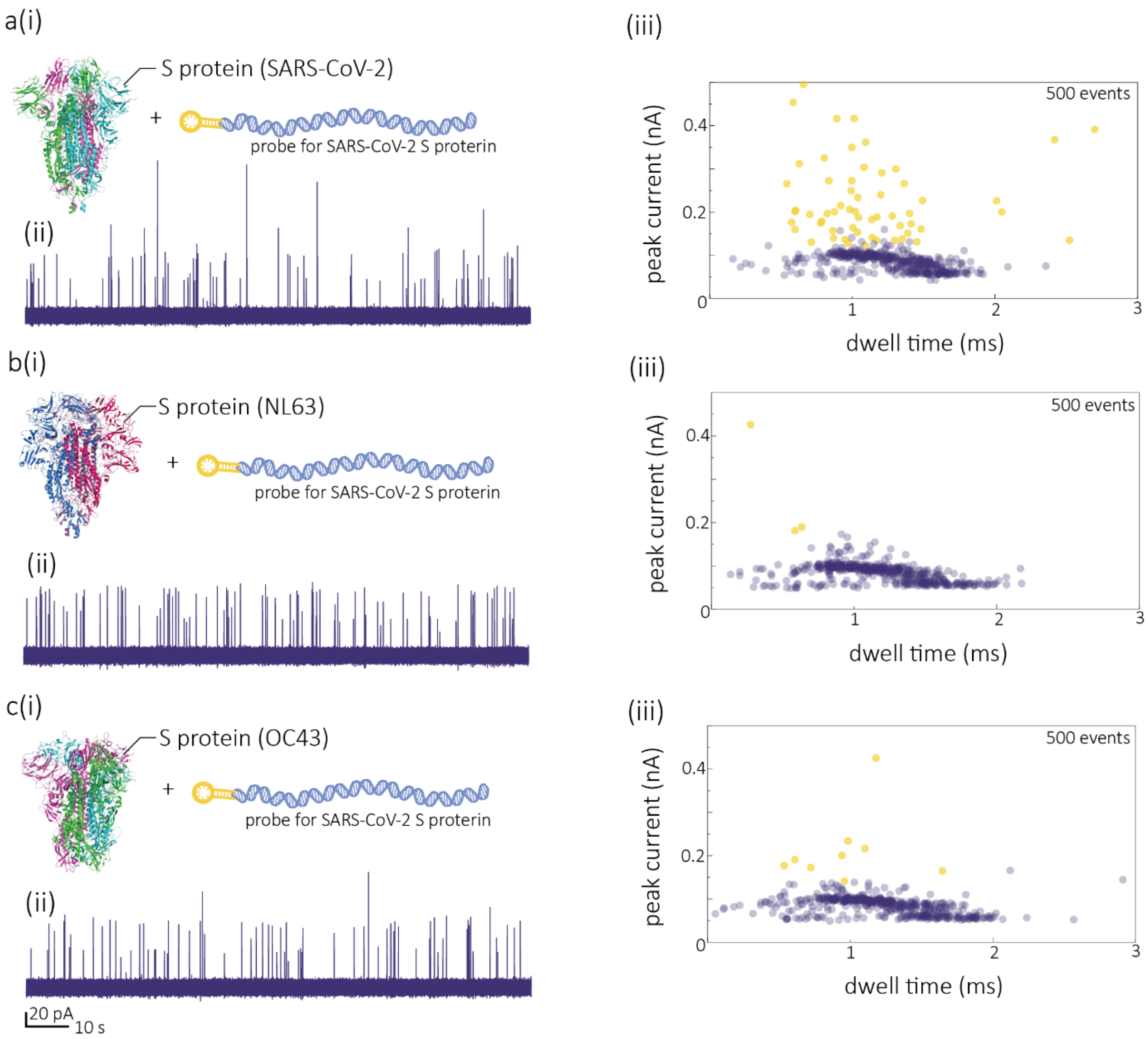

Supplementary Fig. 10 | Control experiments with S protein associated with seasonal flu viruses.

Representative current-time traces for the SBA-labelled molecular probes translocations in the presence of S protein from **(a)** SARS-CoV-2, **(b)** seasonal flu virus NL63, and **(c)** seasonal flu virus OC43. Schematics for the proteins and DNA probes are shown in (i), and scatter plots of peak current versus dwell time for 500 events are shown in (iii), with protein-bound events highlighted in yellow. All the translocation experiments were performed using molecular probes at a concentration of 200 pM and 20 nM protein in 2 M LiCl buffer (5 mM $MgCl_2$, 10 mM Tris-HCl, 1 mM EDTA, pH = 8) at an applied potential bias of 300 mV.

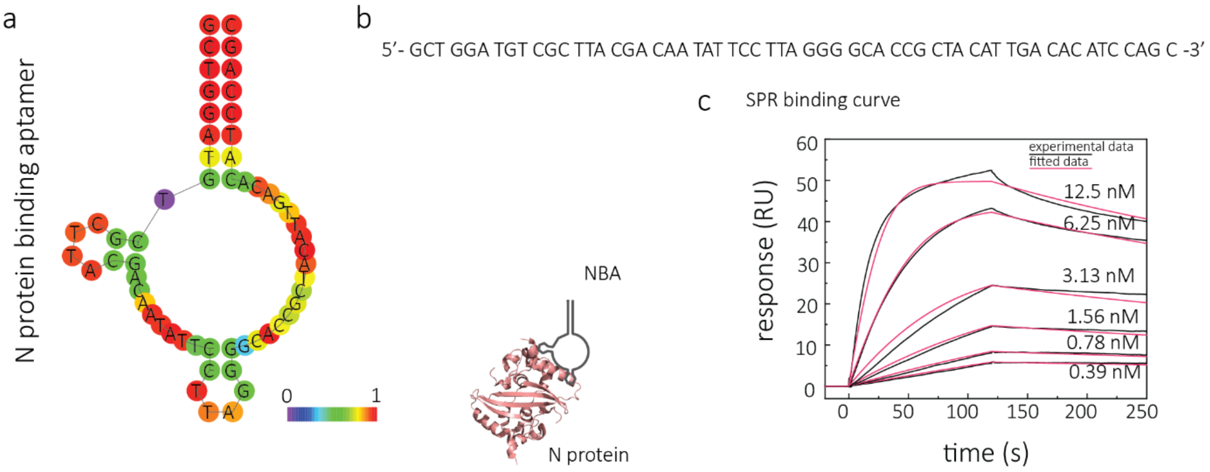

Supplementary Fig. 11 | NBA aptamer and its binding to the N protein of SARS-CoV-2.

(a) Graphical representation showing the predicted structure of NBA aptamer using the centroid secondary structure. Colour bar represents the predicted probability using the lowest free energy. The graphical representation of the N protein and its binding to NBA is shown on the right side. **(b)** Sequence of NBA. **(c)** SPR response curves of NBA upon the addition of N protein at concentrations of 0.39, 0.78, 1.56, 3.13, 6.25, and 12.5 nM, respectively. The black lines are experimental measurement data, and the red lines are fitted data. The K_D for this NBA to the N protein was calculated to be 0.73 nM. Source data are provided as a Source Data file.

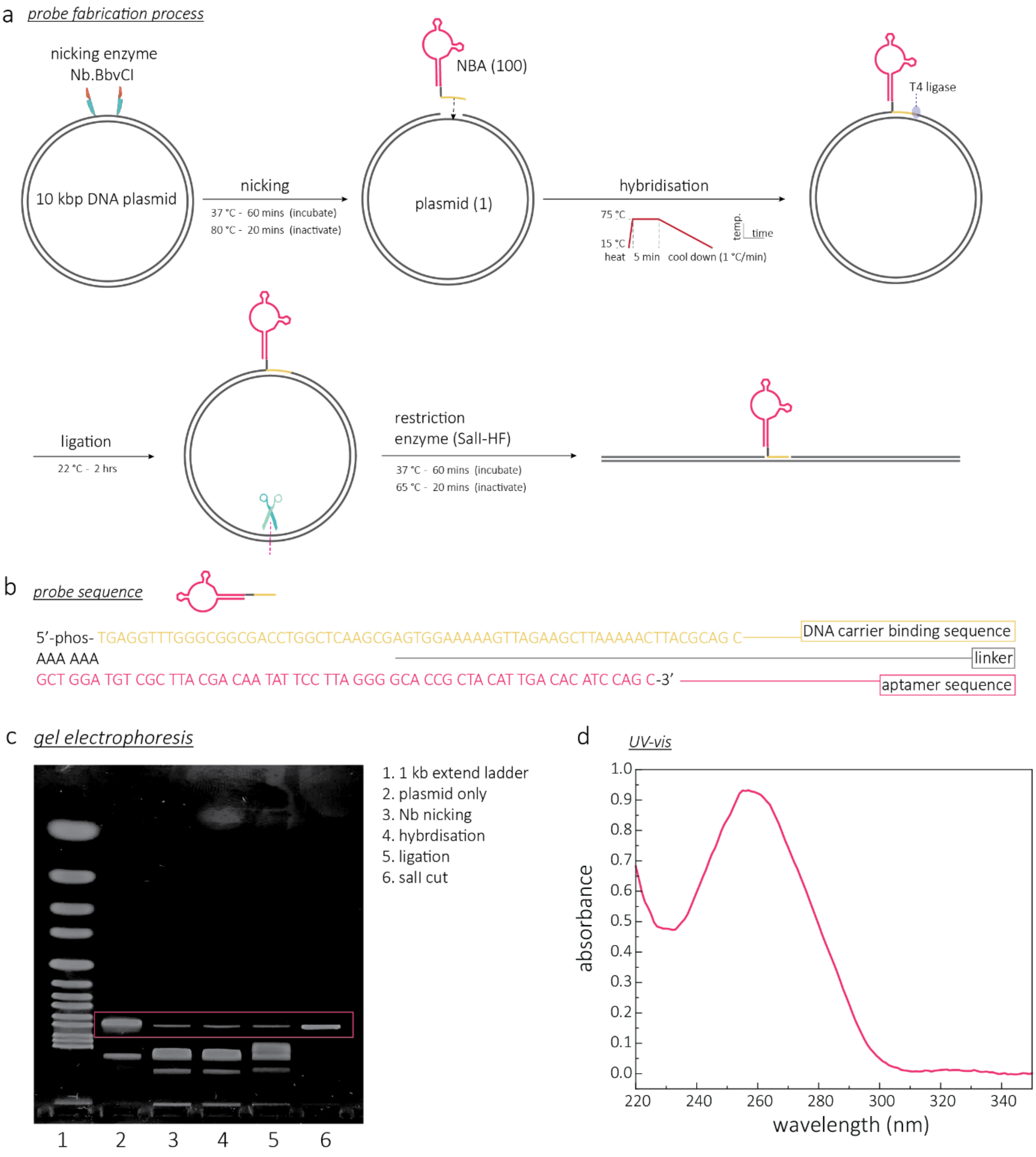

Supplementary Fig. 12 | Preparation and characterisation of NBA-labelled DNA molecular probes.

(a) Schematic illustration of the preparation process of the NBA-labelled molecular probe from a 10 kbp DNA plasmid. **(b)** Schematic and sequence of NBA probe used for modification of molecular probe. The probe consists of three parts: the DNA carrier binding sequence (yellow), the 6-A linker (black), and the aptamer sequence (red). **(c)** Gel electrophoresis characterisation for each step of the preparation process of NBA-labelled molecular probe. **(d)** UV-Vis spectrum for the DNA molecular probe extracted from gel electrophoresis. The concentration of the molecular probe was determined using the absorbance at 260 nm. Source data are provided as a Source Data file.

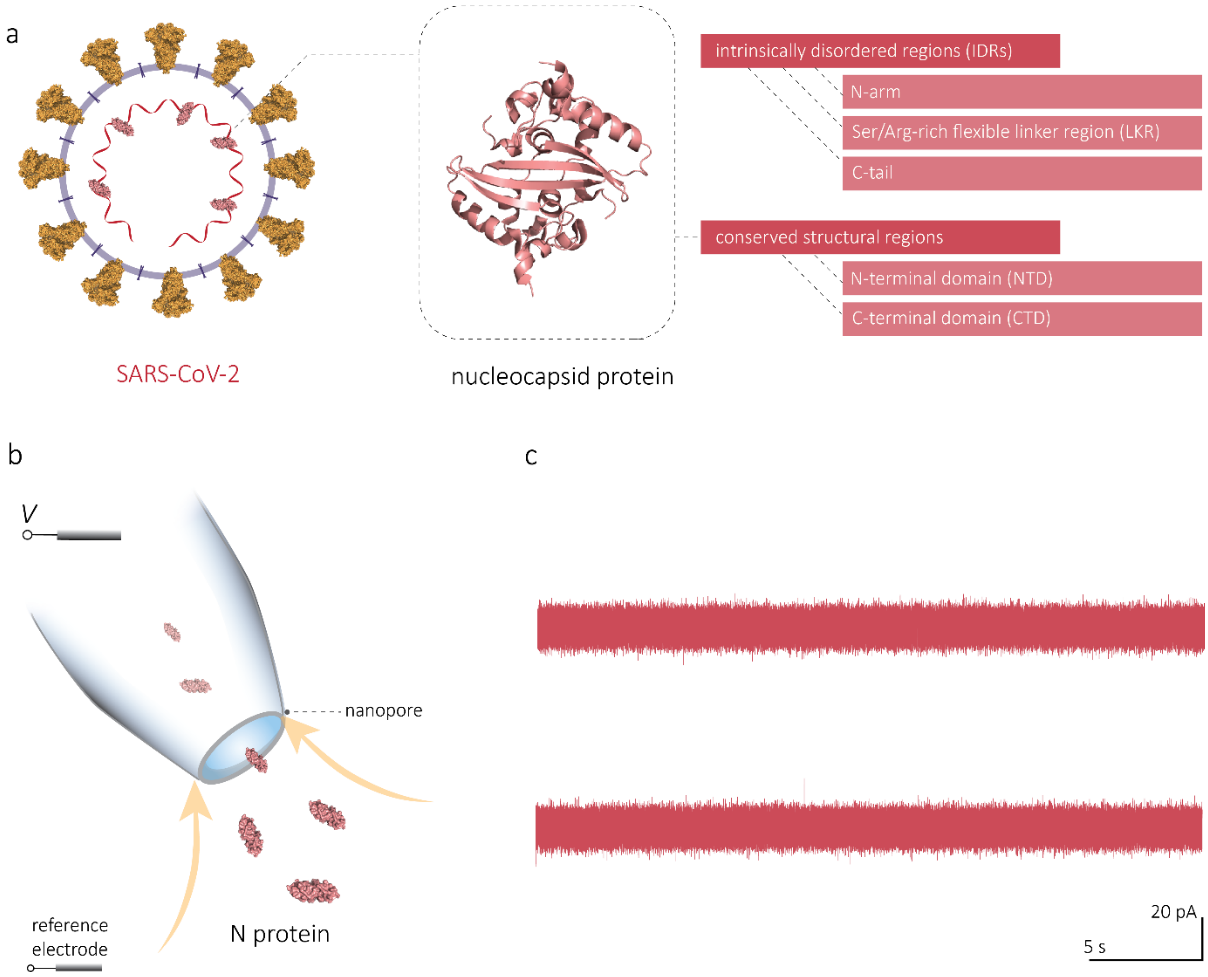

Supplementary Fig. 13 | Model of the N protein along with Current time trace.

(a) Schematic representation of SARS-CoV-2 and nucleocapsid protein structure and its composition. **(b)** Schematic for the translocation of N protein (20 nM) through a nanopore. **(c)** Representative current-time traces for the translocation of N protein. No translocation events can be detected. Translocation experiments were performed with 20 nM N protein in 2 M LiCl buffer (5 mM $MgCl_2$, 10 mM Tris-HCl, 1 mM EDTA, pH = 8) at an applied potential bias of 300 mV.

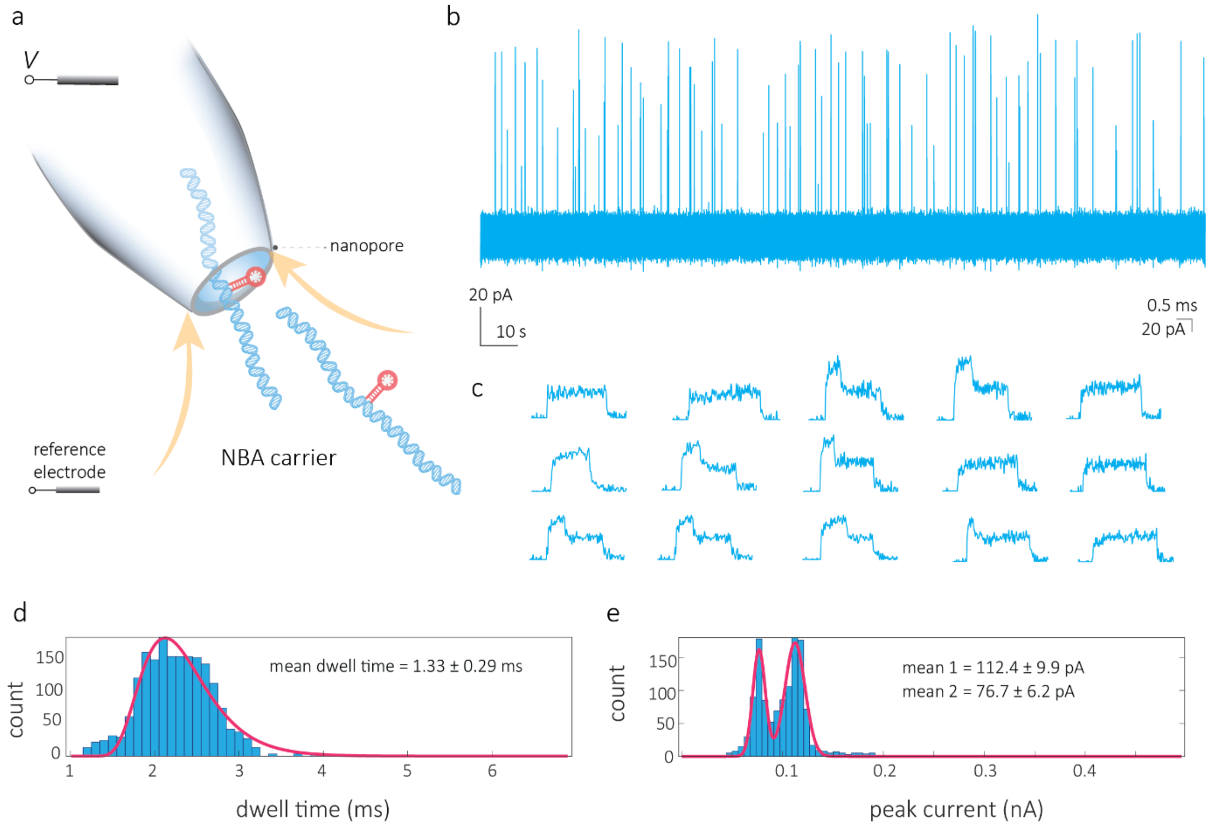

Supplementary Fig. 14 | Translocation of NBA-labelled DNA molecular probes only.

Schematic **(a)** and representative current-time trace **(b)** showing the NBA-labelled molecular probe (10 kbp) translocating through a nanopore in the absence of target proteins. **(c)** Zoom-in view of typical translocation events. **(d)** Histograms of dwell time for the nanopore detection of NBA-labelled molecular probes with mean dwell time of 1.33 ± 0.29 ms. **(e)** Histograms of peak current for the translocations. Two distinct peaks were observed at 76.7 ± 6.2 pA (linear) and 112.4 ± 9.9 pA (folded), respectively, indicating the linear and folded state of DNA. All the translocation experiments were performed with 200 pM molecular probes in 2 M LiCl buffer (5 mM $MgCl_2$, 10 mM Tris-HCl, 1 mM EDTA, pH = 8) at an applied potential bias of 300 mV.

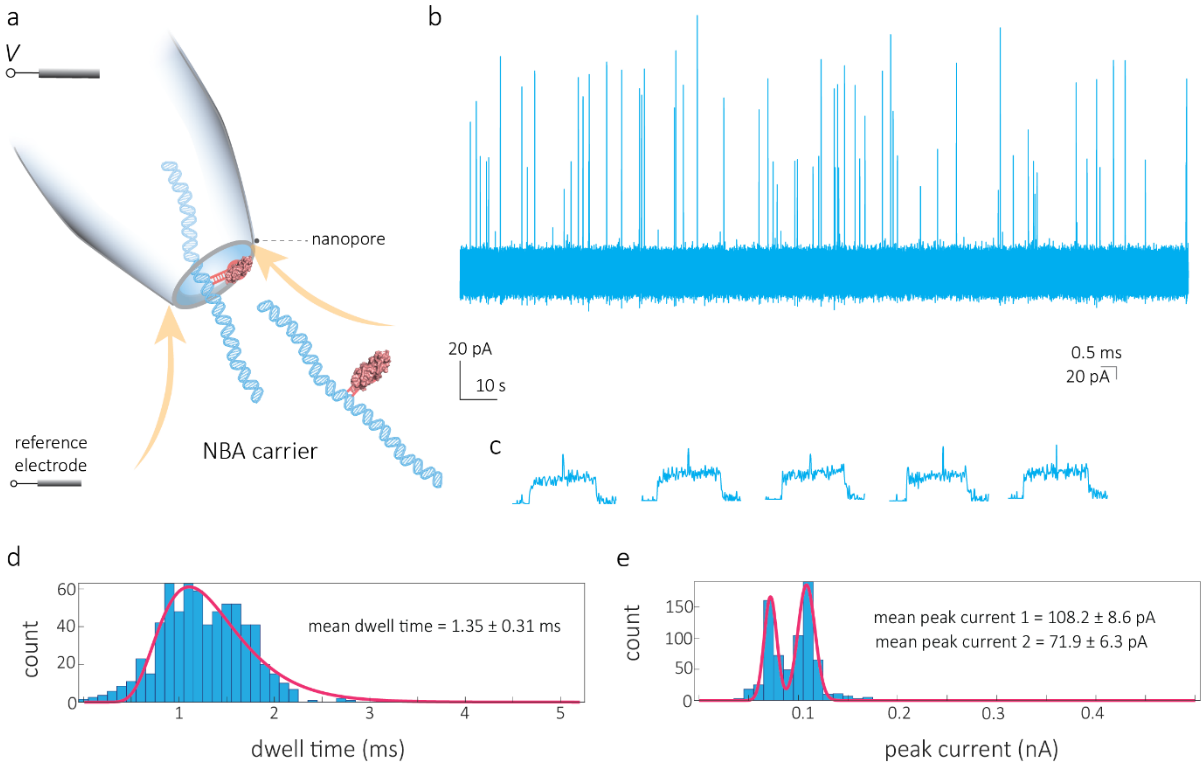

Supplementary Fig. 15 | Translocation of NBA-labelled DNA molecular probes bound to N protein.

Schematic **(a)** and representative current-time trace **(b)** showing the NBA-labelled molecular probe (200 pM) translocating through a nanopore in the presence of N protein (20 nM). **(c)** Zoom-in view of typical translocation events for N protein bound to NBA-labelled molecular probe. **(d)** Histograms of dwell time (1.35 ± 0.31 ms) for the nanopore detection of NBA-labelled molecular probes with N protein. **(e)** Histograms of peak current for the translocations. Two peaks were observed at 71.9 ± 6.3 pA and and 108.2 ± 8.6 pA, respectively. All translocation experiments were performed with 200 pM molecular probes in 2 M LiCl buffer (5 mM $MgCl_2$, 10 mM Tris-HCl, 1 mM EDTA, pH = 8) at an applied potential bias of 300 mV.

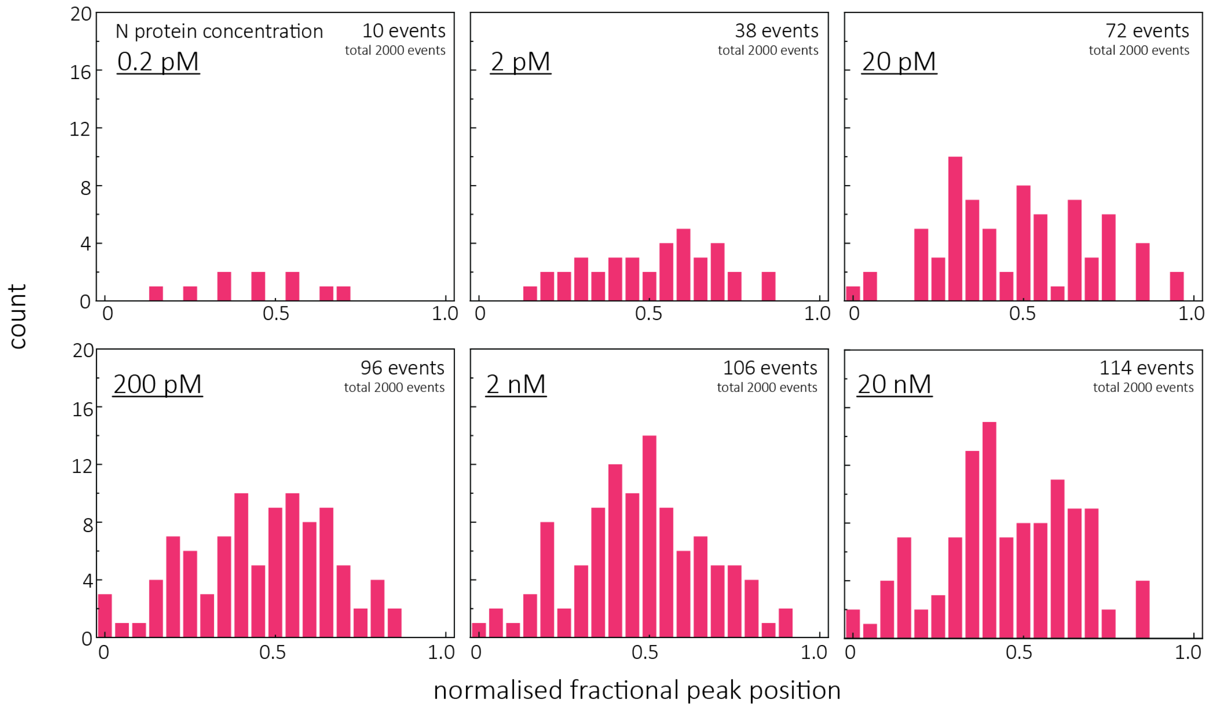

Supplementary Fig. 16 | Concentration dependence of N protein on the fractional position.

Translocation events were detected with middle sub-peaks for the NBA-labelled molecular probe in the presence of 0.2 pM, 2 pM, 20 pM, 200 pM, 2 nM and 20 nM of N protein. All translocation experiments were performed using 200 pM NBA-labelled molecular probes in 2 M LiCl buffer (5 mM $MgCl_2$, 10 mM Tris-HCl, 1 mM EDTA, pH = 8) at an applied potential bias of 300 mV. Source data are provided as a Source Data file.

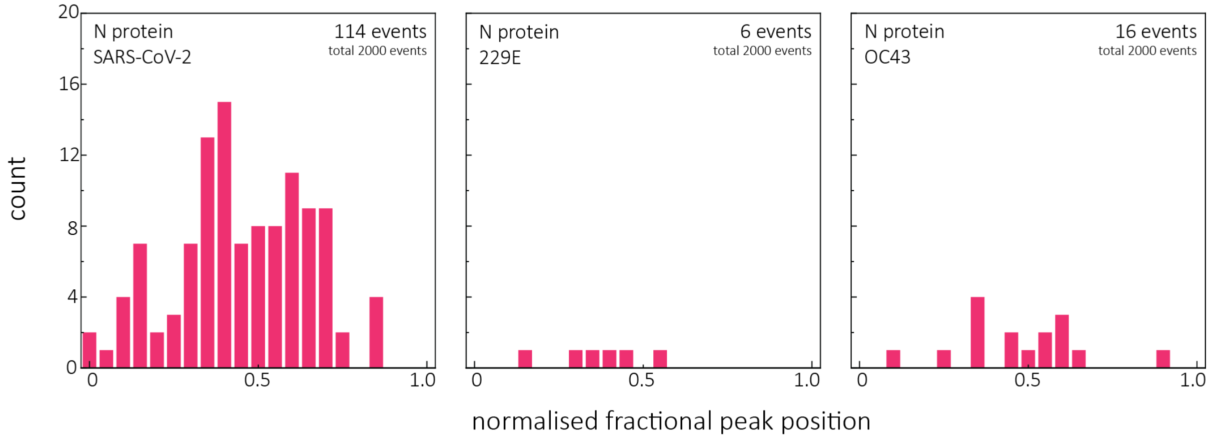

Supplementary Fig. 17 | Control experiments for determination of the fractional position of N protein associated with seasonal flu viruses.

Translocation events were detected with middle sub-peaks for the NBA-labelled molecular probe in the presence of N protein for SARS-CoV-2, seasonal flu virus 229E, and seasonal flu virus OC43. A total of 2000 translocation events were analysed for each experiment. All the translocation experiments were performed using 200 pM NBA-labelled molecular probes in 2 M LiCl buffer (5 mM $MgCl_2$, 10 mM Tris-HCl, 1 mM EDTA, pH = 8) at an applied potential bias of 300 mV. Source data are provided as a Source Data file.

a

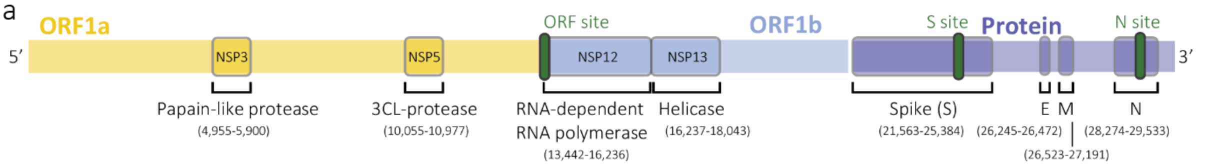

b

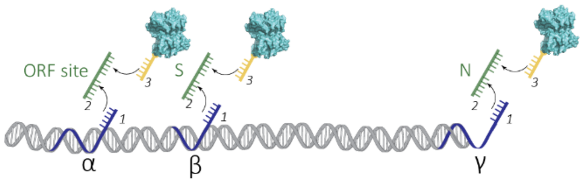

	18% position	48% position	100% position
probe (protein side)	α3	β3	γ3
amplified target	α2	β2	γ2
probe (carrier side)	α1	β1	γ1

c

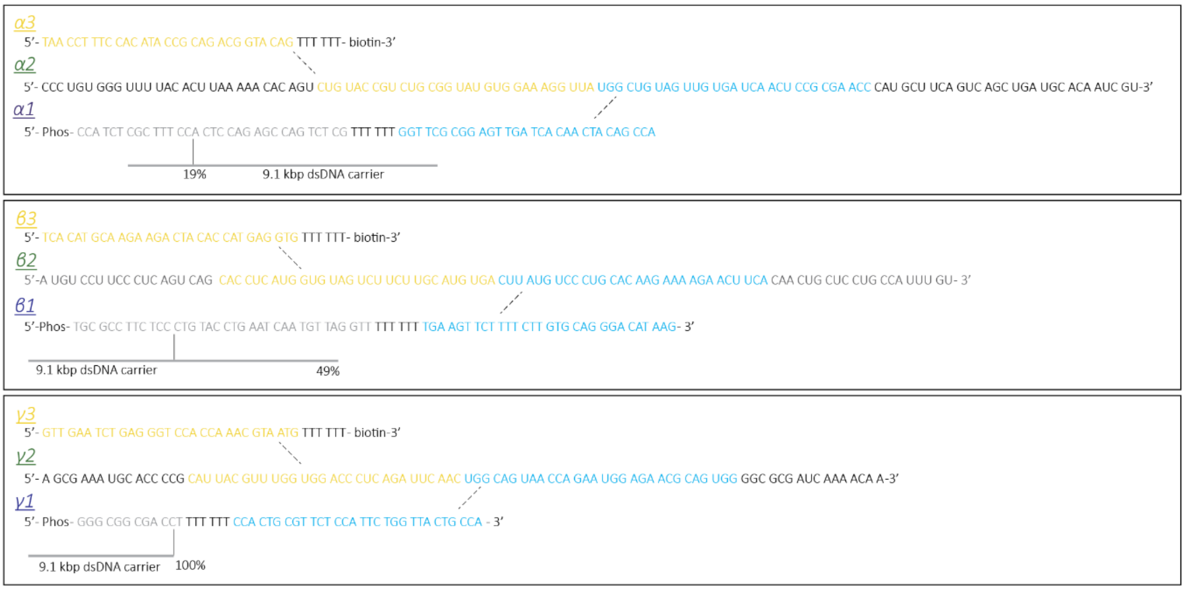

Supplementary Fig. 18 | Design for multiplexed detection of SARS-CoV-2 RNA.

(a) Genome map showing the full length with annotated regions of SARS-CoV-2. The relative positions of ORF, S and N genes selected to be detected are highlighted in green. **(b)** Schematic representations of a 3-site DNA molecular probe (9.1 kbp) and the binding to ORF, S, and N gene targets, respectively. The probe for an individual target (α1, β1, γ1) is assigned to a specific position along the dsDNA (α, β, γ) and bound to the first half of the target RNA sequence (α2, β2, γ2). A biotinylated sequence (α3, β3, γ3) is used as the reporting probe to bind the second half of the target sequence (α2, β2, γ2) and streptavidin to enhance the signal. The right-hand side table summarises the relative position of the selected ORF, S, and N gene targets. Sequences of these probes and targets for each position are shown in **(c)**.

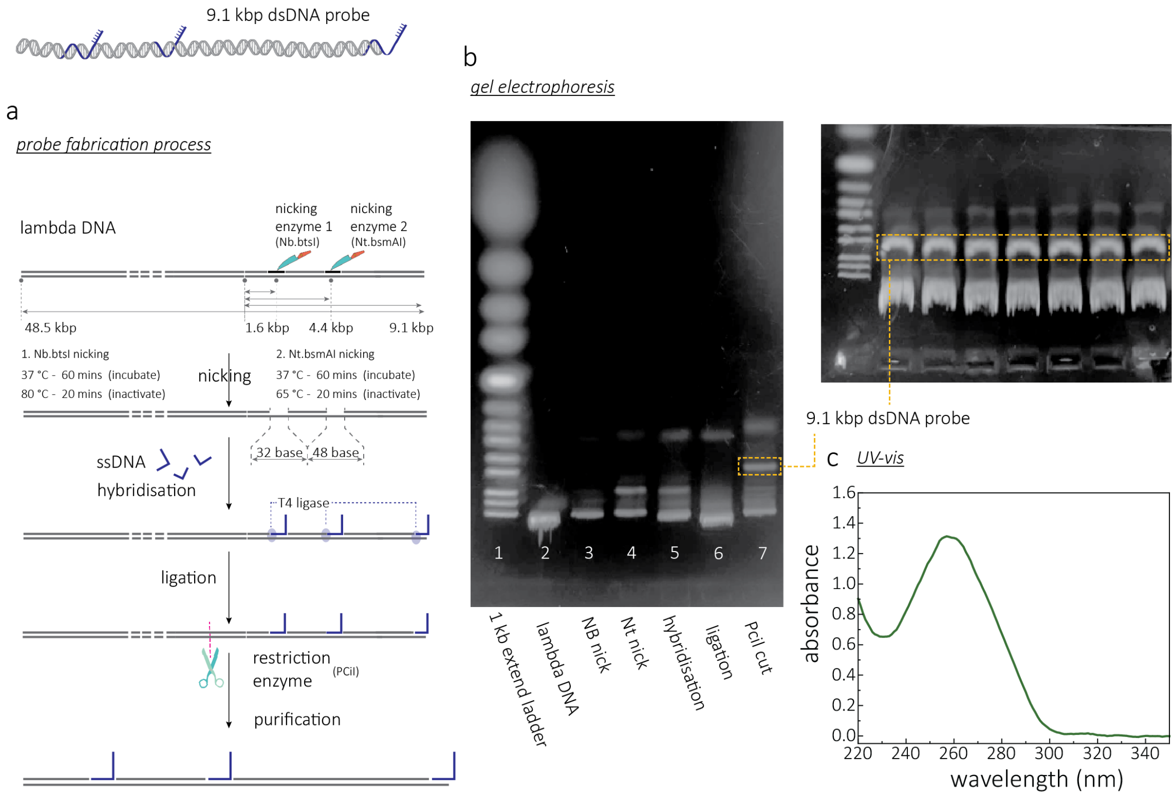

Supplementary Fig. 19 | Preparation and characterisation for a 3-site molecular probe (9.1 kbp).

(a) Schematic illustration of the preparation process of the 3-site molecular probe (9.1 kbp) obtained from λ-DNA. **(b)** A gel electrophoresis characterisation for each step of the preparation process of the 3-site molecular probe (9.1 kbp). It is worth noting that a band of 5.3 to 5.6 kbp fragment can be observed after the nicking step (lanes 3 to 7). This fragment is generated by two relatively close nicking sites (on opposite strands) along lambda DNA. There is no overlap with the 9.1 kbp target fragment, and it can be distinguished from the target band (lane 7). A preparation gel on the right-hand side shows a clear band of the target 9.1 kbp molecular probe. **(c)** UV-Vis spectrum for the molecular probe extracted from gel electrophoresis. The concentration of the DNA molecular probe was determined using the absorbance at 260 nm. Source data are provided as a Source Data file.

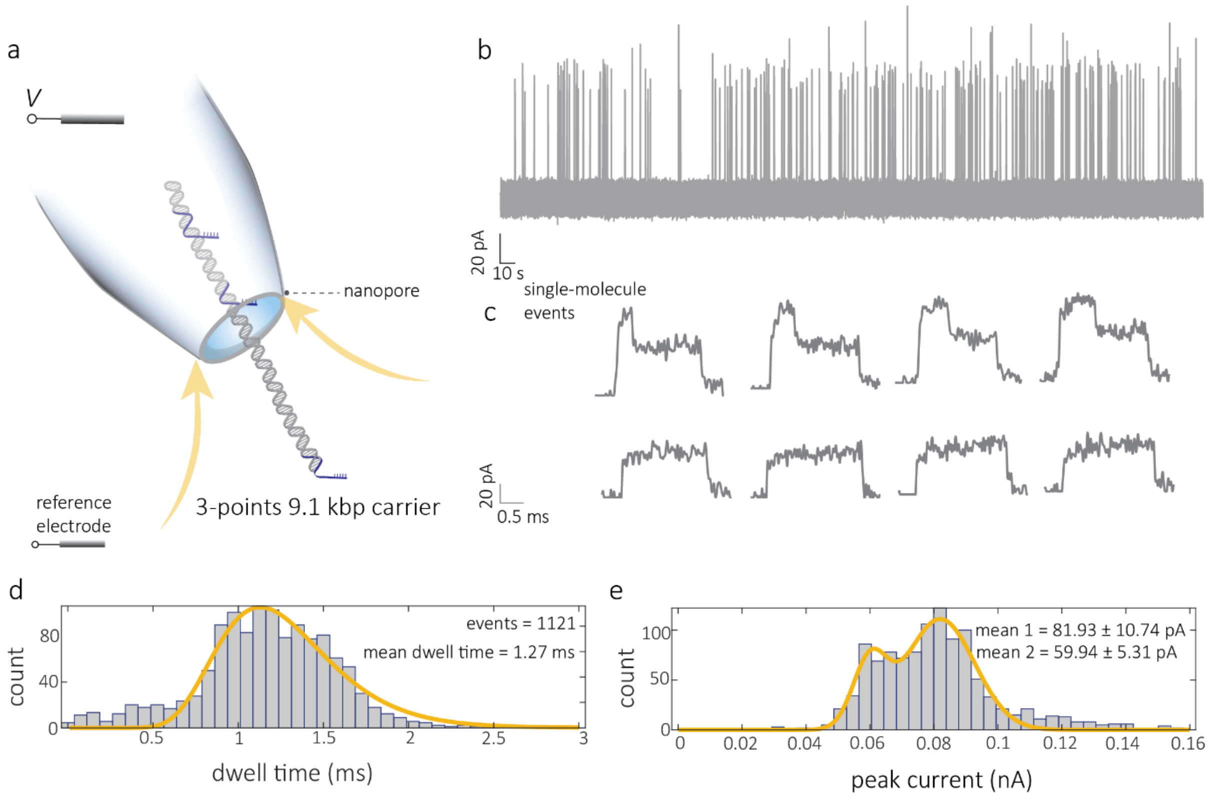

Supplementary Fig. 20 | Translocation of 3-site 9.1 kbp molecular probe only.

Schematic **(a)** and representative current-time trace **(b)** showing the 3-site molecular probe (9.1 kbp) translocating through a nanopore in the absence of target RNA. **(c)** zoom-in view of typical translocation events showing the molecular probe translocating linearly or in folded-state. **(d)** Histograms of dwell time for the 3-site 9.1 kbp molecular probes with mean dwell time of 1.27 ± 0.37 ms (n = 1121). **(e)** Histograms of peak current for the translocations (n = 1121). Two distinct populations were observed, at 59.9 ± 5.3 pA and at 81.9 ± 10.7 pA, respectively, indicating the linear and folded state of DNA. All the translocation experiments were performed using 200 pM molecular probes in 2 M LiCl buffer (5 mM $MgCl_2$, 10 mM Tris-HCl, 1 mM EDTA, pH = 8) at an applied potential bias of 300 mV.

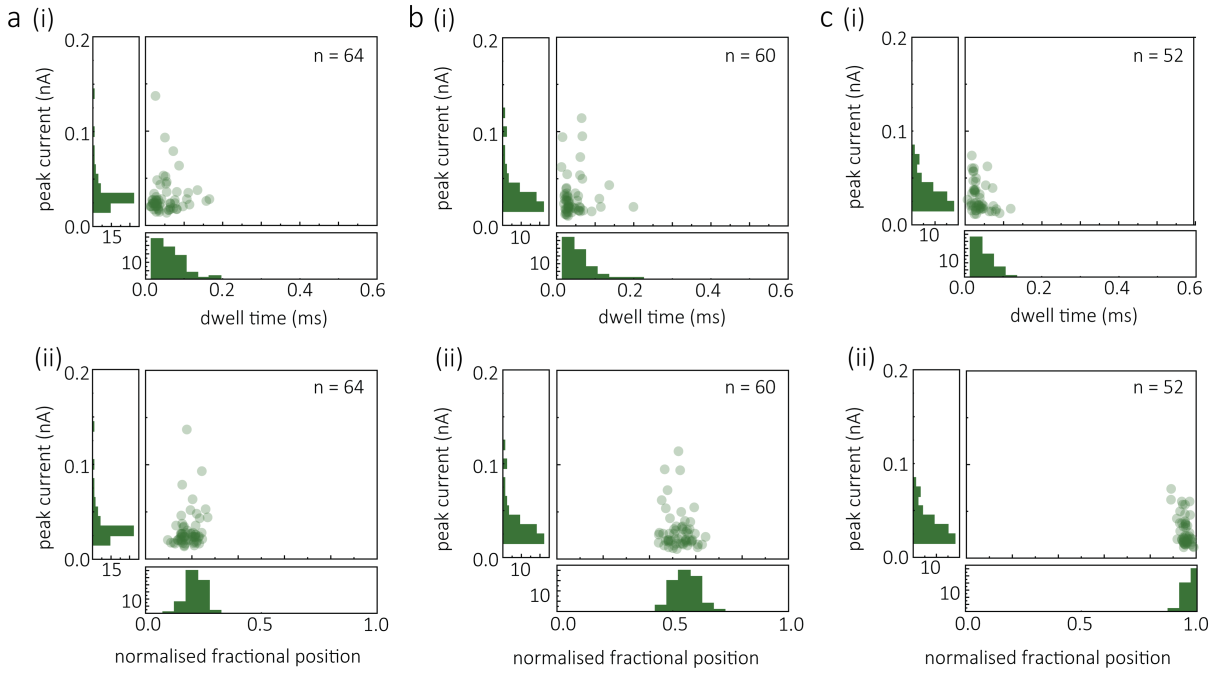

Supplementary Fig. 21 | Scatter plots for sub-peaks detected at fractional positions 0.2, 0.5 and 1.0

Scatter plots of (i) subpeak current vs subpeak dwell time, (ii) subpeak current vs normalised fractional peak position for (a) ORF1b, (b) S, and (c) N gene targets on the 3-site DNA molecular probe (9.1 kbp). Source data are provided as a Source Data file.

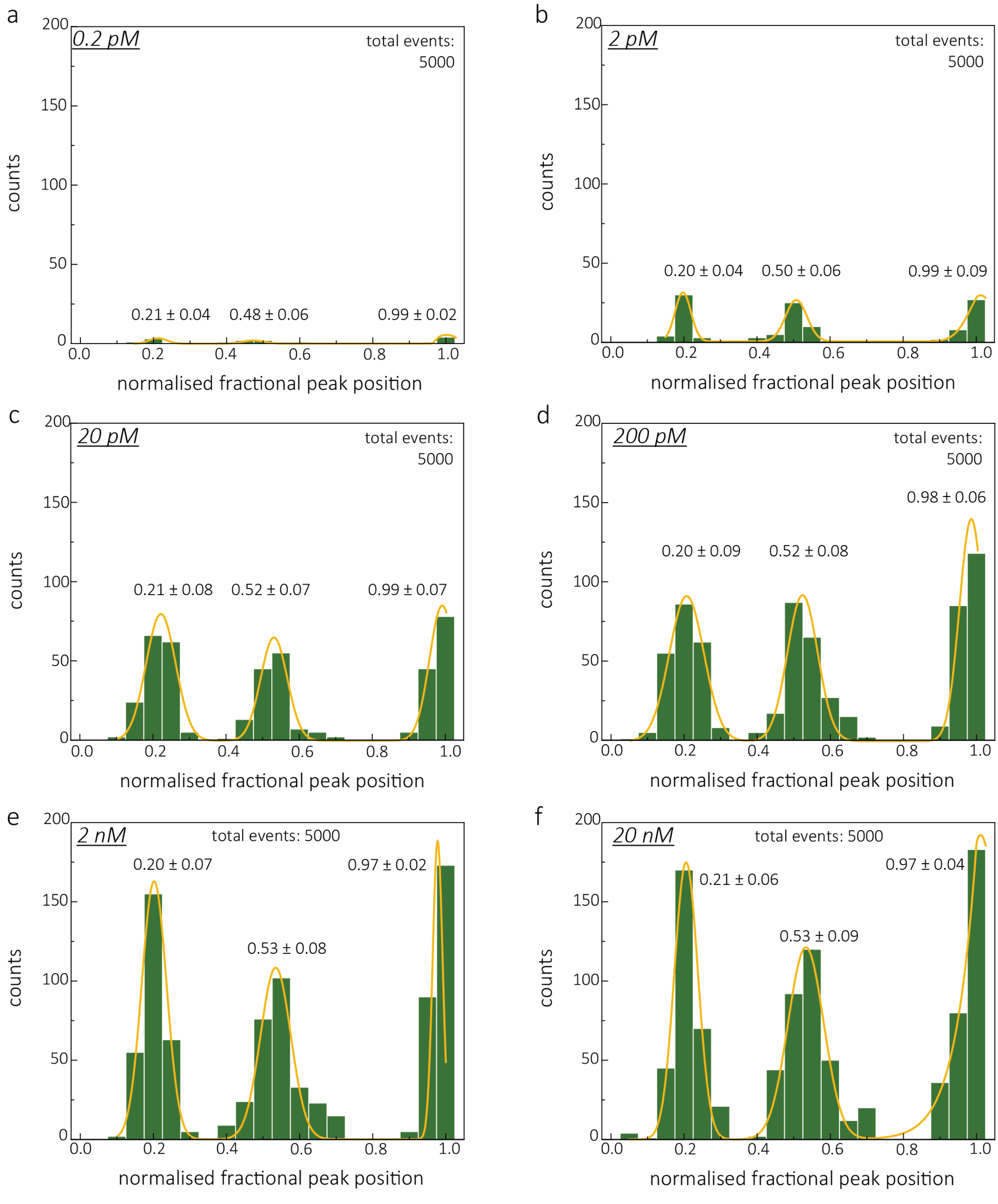

Supplementary Fig. 22 | Concentration dependence of synthetic RNA detection.

Translocation events detected with sub-peak at respective fractional positions for the 3-site DNA molecular probe in the presence of **(a)** 0.2 pM, **(b)** 2 pM, **(c)** 20 pM, **(d)** 200 pM, **(e)** 2 nM, and **(f)** 20 nM of synthetic RNA targets (analogue to ORF, S and N gene sequences). A total of 5000 translocation events were used for analysis of the data. All fractional positions were normalised and fitted with a Gaussian function. All translocation experiments were performed with 200 pM 3-site molecular probe in 2 M LiCl buffer (5 mM $MgCl_2$, 10 mM Tris-HCl, 1 mM EDTA, pH = 8) at an applied potential bias of 300 mV.

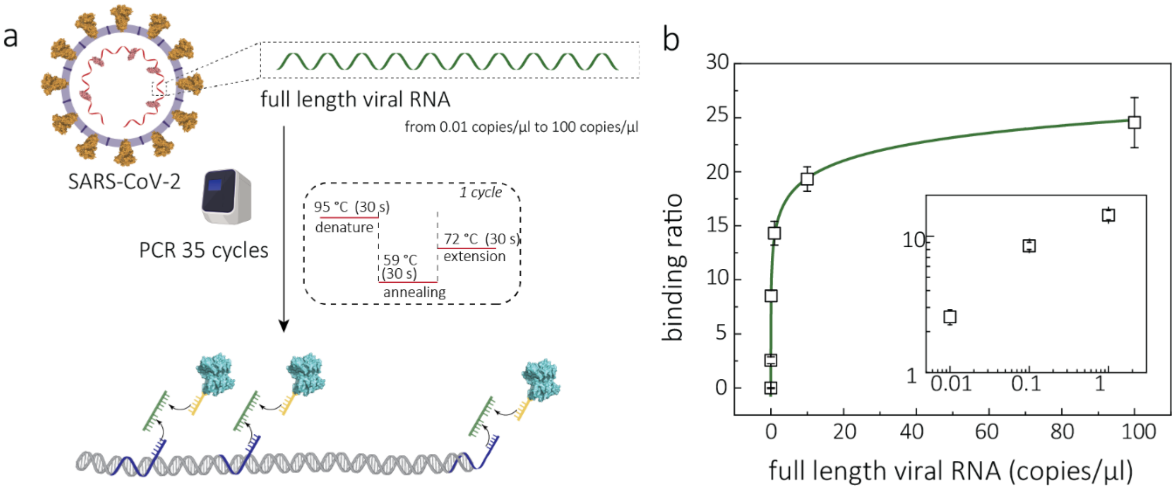

Supplementary Fig. 23 | Detection of full-length SARS-CoV-2 RNA.

(a) Schematic showing the PCR amplification of a specific region of full-length SARS-CoV-2 RNA. The amplification was run for 35 cycles before nanopore testing. **(b)** Response of binding ratio over the increasing copies numbers per microlitre from 0.01 to 100 copies/ µl. Inset shows a linear response from 0.01 to 1 copies/µl of full-length viral RNA. All the translocation experiments were performed using 200 pM molecular probes in 2 M LiCl buffer (5 mM $MgCl_2$, 10 mM Tris-HCl, 1 mM EDTA, pH = 8) at an applied potential bias of 300 mV. Error bars in **b** represent the standard deviation of three independent replicates, and the measure of the centre represents their corresponding mean value. Source data are provided as a Source Data file.

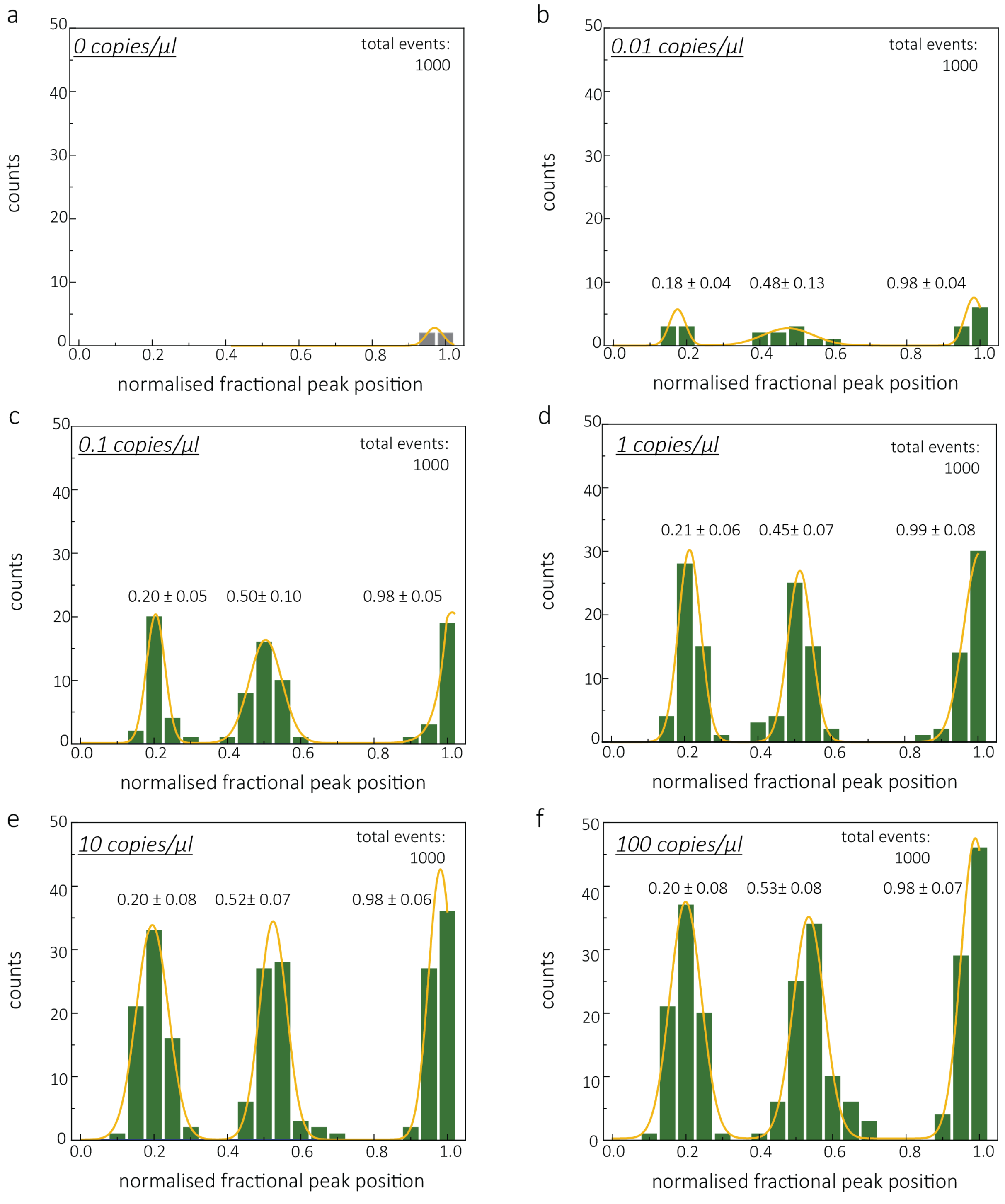

Supplementary Fig. 24 | Concentration dependence of full-length SARS-CoV-2 RNA detection.

Translocation events detected with sub-peak at respective fractional positions for the 3-site molecular probe in the presence of **(a)** 0 copies/μl, **(b)** 0.01 copies/μl, **(c)** 0.1 copies/μl, **(d)** 1 copies/μl, **(e)** 10 copies/μl, and **(f)** 100 copies/μl of full-length SARS-CoV-2 RNA amplified targets (ORF, S and N gene). A total of 1000 translocation events were used to analyse the results for each concentration. All the fractional positions were normalised and fitted with a Gaussian function. All the translocation experiments were performed with 200 pM molecular probes in 2 M LiCl buffer (5 mM $MgCl_2$, 10 mM Tris-HCl, 1 mM EDTA, pH = 8) at an applied potential bias of 300 mV.

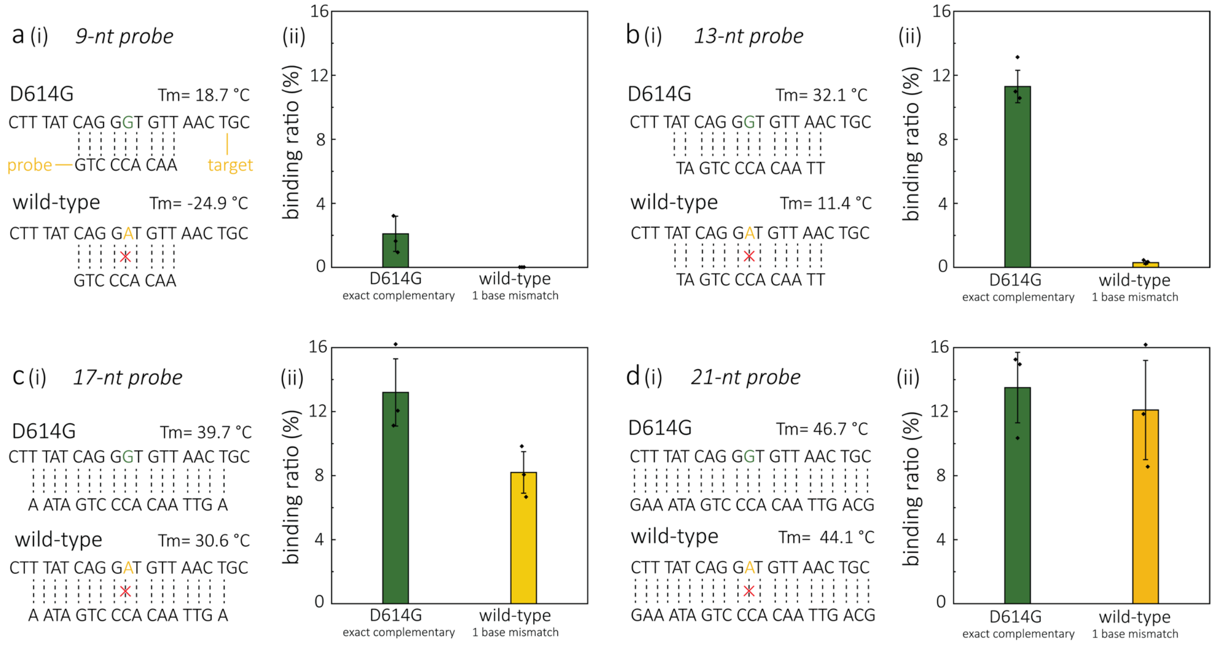

Supplementary Fig. 25 | Optimisation of probe length for discriminating the D614G mutation.

Detection of the D614G mutation using a designed DNA molecular probe with receptor lengths of 9-nt **(a)**, 13-nt **(b)**, 17-nt **(c)**, and 21-nt **(d)**. Binding and predicted melting temperature (Tm) of designed probes towards the wild-type and D614G genes are shown in (i). Incubation and nanopore experiments were performed at room temperature (22 °C). Corresponding binding ratios are shown in (ii). The 13-nt probe has the highest potential to differentiate the D614G mutant and wild-type genes, with the most significant difference between binding ratios. Source data are provided as a Source Data file.

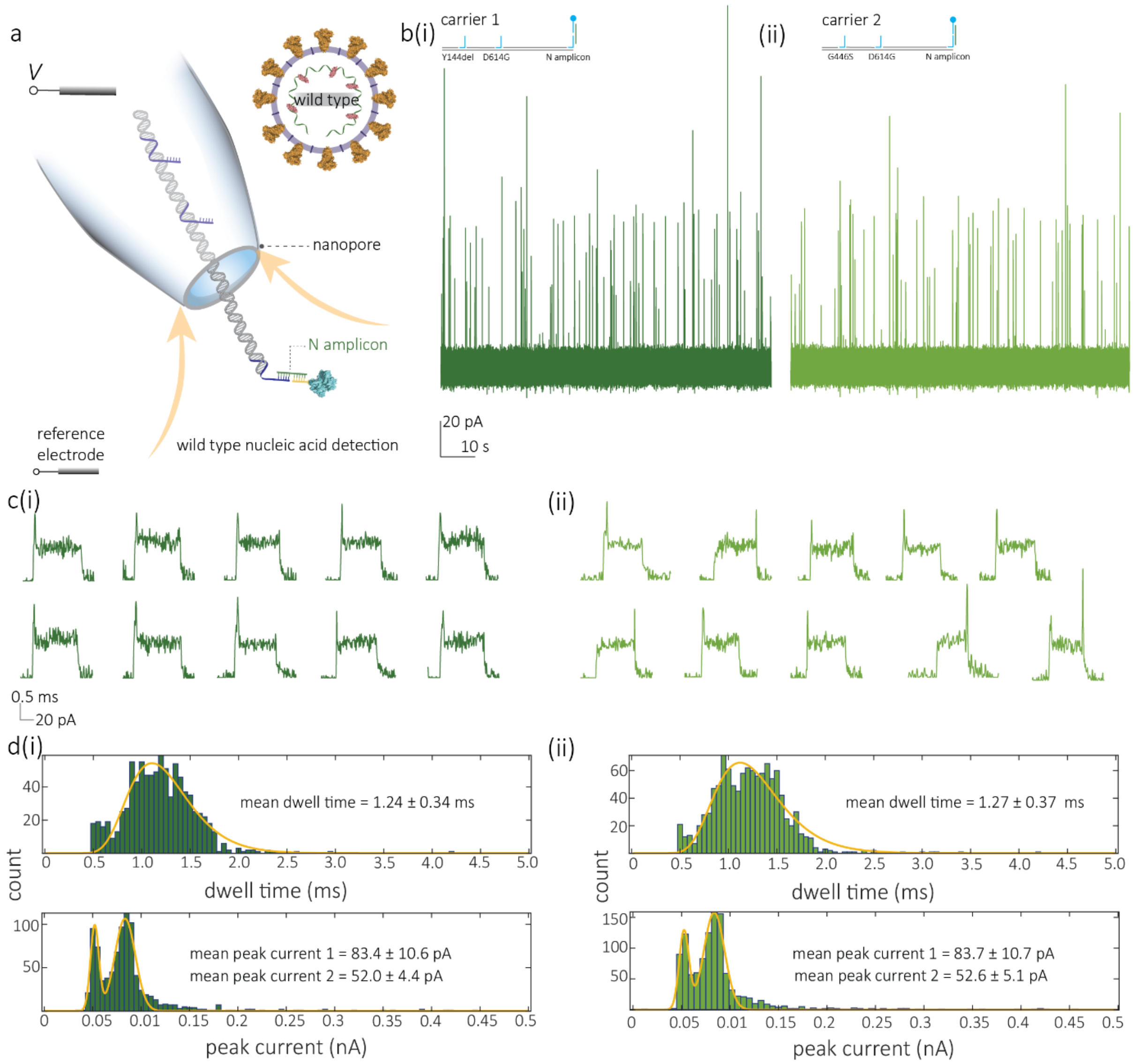

Supplementary Fig. 26 | Testing wild-type SARS-CoV-2 with mutation-specific DNA molecular probes.

(a) Schematic showing the translocation of molecular probes edited with probes specific for mutations of Y114del/G446S and D614G, and probe for N gene. Probe 1 was edited with probes specific for mutation of Y114del and D614G at sites 0.18 and 0.48 and probe for N gene at site 1. Probe 2 was edited for G446S, D614G, and N genes at sites 0.18, 0.48, and 1. For wild-type SARS-CoV-2 RNA, only the N gene can be detected with sub-peak observed in the position of 1 for either Probe 1 or 2. **(b)** Representative current-time traces for Probe 1 (i) and Probe 2 (ii) translocating through a nanopore in the presence of RNA amplicons of full-length wild-type SARS-CoV-2 RNA. **(c)** Zoom-in views of typical binding events for Probe 1 (i) and Probe 2 (ii) showing the N gene amplicon binding in the end (fractional position of 1). **(d)** Histograms of dwell time and peak current for the translocation of Probe 1 (i) and Probe 2 (ii) in the presence of RNA amplicons from wild-type SARS-CoV-2 RNA. Distributions in **(d)** were fitted with Gaussian functions. All the translocation experiments were performed using 200 pM molecular probes in 2 M LiCl buffer (5 mM $MgCl_2$, 10 mM Tris-HCl, 1 mM EDTA, pH = 8) at an applied potential bias of 300 mV.

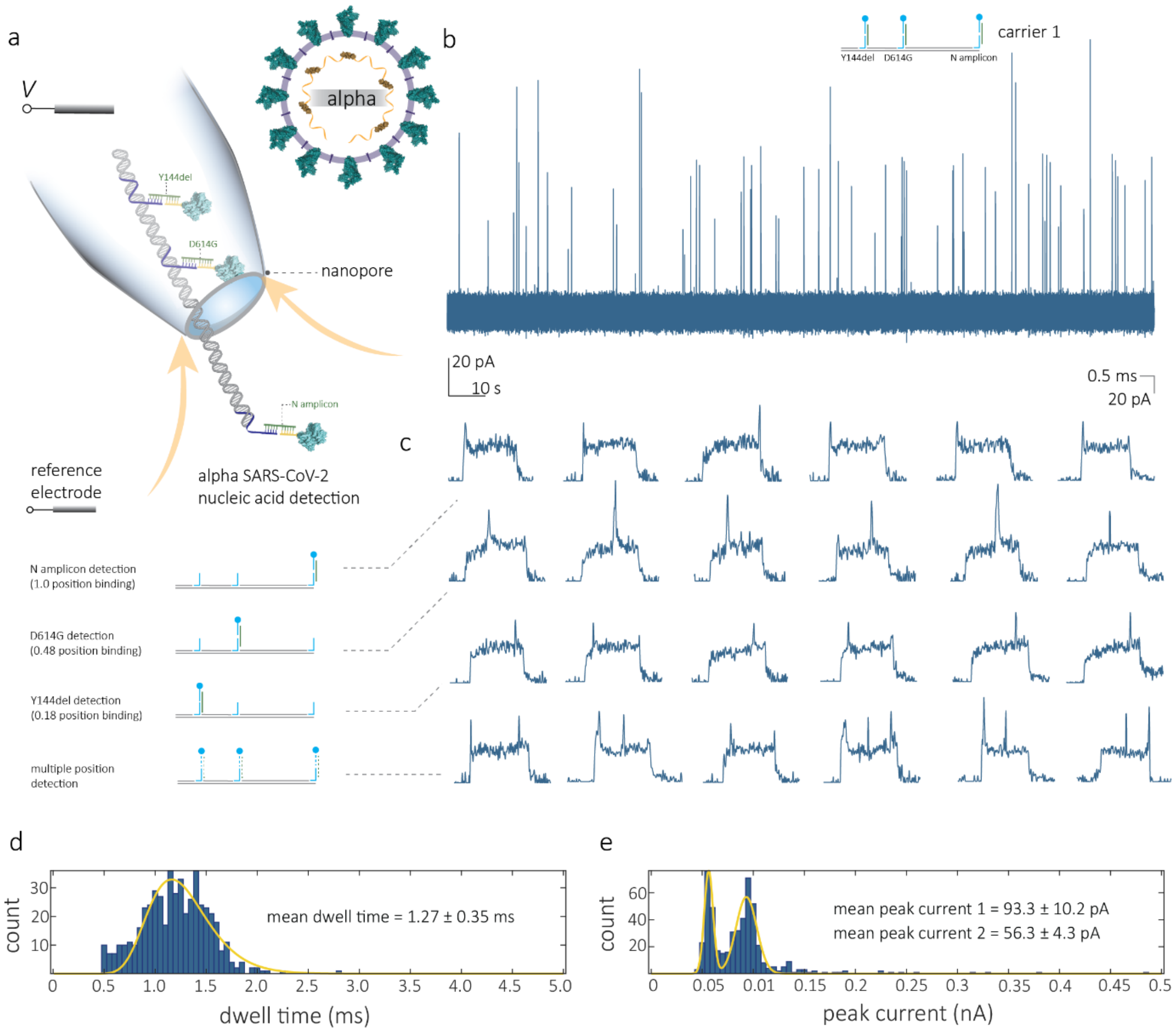

Supplementary Fig. 27 | Test with Alpha variant of SARS-CoV-2.

(a) Schematic showing the translocation of DNA molecular probe (Probe 1) in the presence of Alpha variant of SARS-CoV-2 RNA amplicons. **(b)** Representative current-time trace for the translocation of Probe 1 with Alpha variant of SARS-CoV-2 RNA amplicons (with the region of Y114del, D614G, and N gene). **(c)** Zoom-in view of typical events representing the bound of RNA fragments of Y114del mutation, D614G mutation, and N gene in all the three sites of 0.18, 0.48 and 1 relative positions. Schematics for the bound of events in respective positions are shown on the left panel. For the Alpha variant, all three areas of RNA can be detected simultaneously. Histograms of translocation dwell time and peak current were summarised in **(d)** and **(e)** with Gaussian function fitting. All the translocation experiments were performed using 200 pM molecular probes in 2 M LiCl buffer (5 mM $MgCl_2$, 10 mM Tris-HCl, 1 mM EDTA, pH = 8) at an applied potential bias of 300 mV.

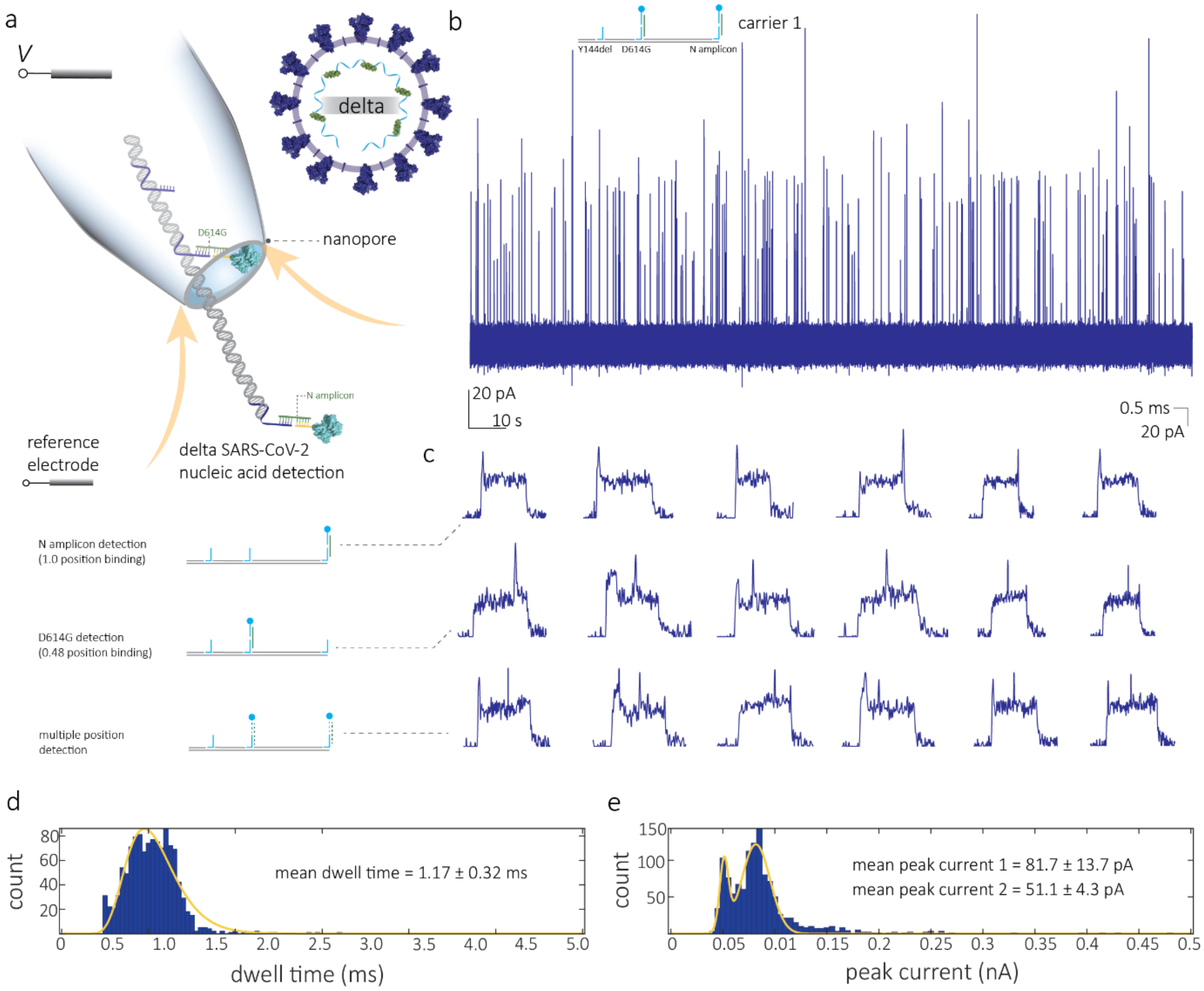

Supplementary Fig. 28 | Test with Delta variant of SARS-CoV-2.

(a) Schematic showing the translocation of molecular probe (Probe 1) in the presence of Delta variant of SARS-CoV-2 RNA amplicons. **(b)** Representative current-time trace for the translocation of Probe 1 with Delta variant of SARS-CoV-2 RNA amplicons (with the region of Y114del, D614G, and N gene). **(c)** Zoom-in view of typical events. As for the amplicon from the Delta variant, two types of sub-peaks at the relative positions of 0.48 and 1 can be observed, representing the presence of the D614G mutation of the N gene fragments. No sub-peak at a position of 0.18 can be observed, which indicates the absence of the Y114del mutation point of the Delta variant. Histograms of translocation dwell time and peak current were summarised in **(d)** and **(e)** with Gaussian function fitting. All the translocation experiments were performed using 200 pM molecular probes in 2 M LiCl buffer (5 mM $MgCl_2$, 10 mM Tris-HCl, 1 mM EDTA, pH = 8) at an applied potential bias of 300 mV.

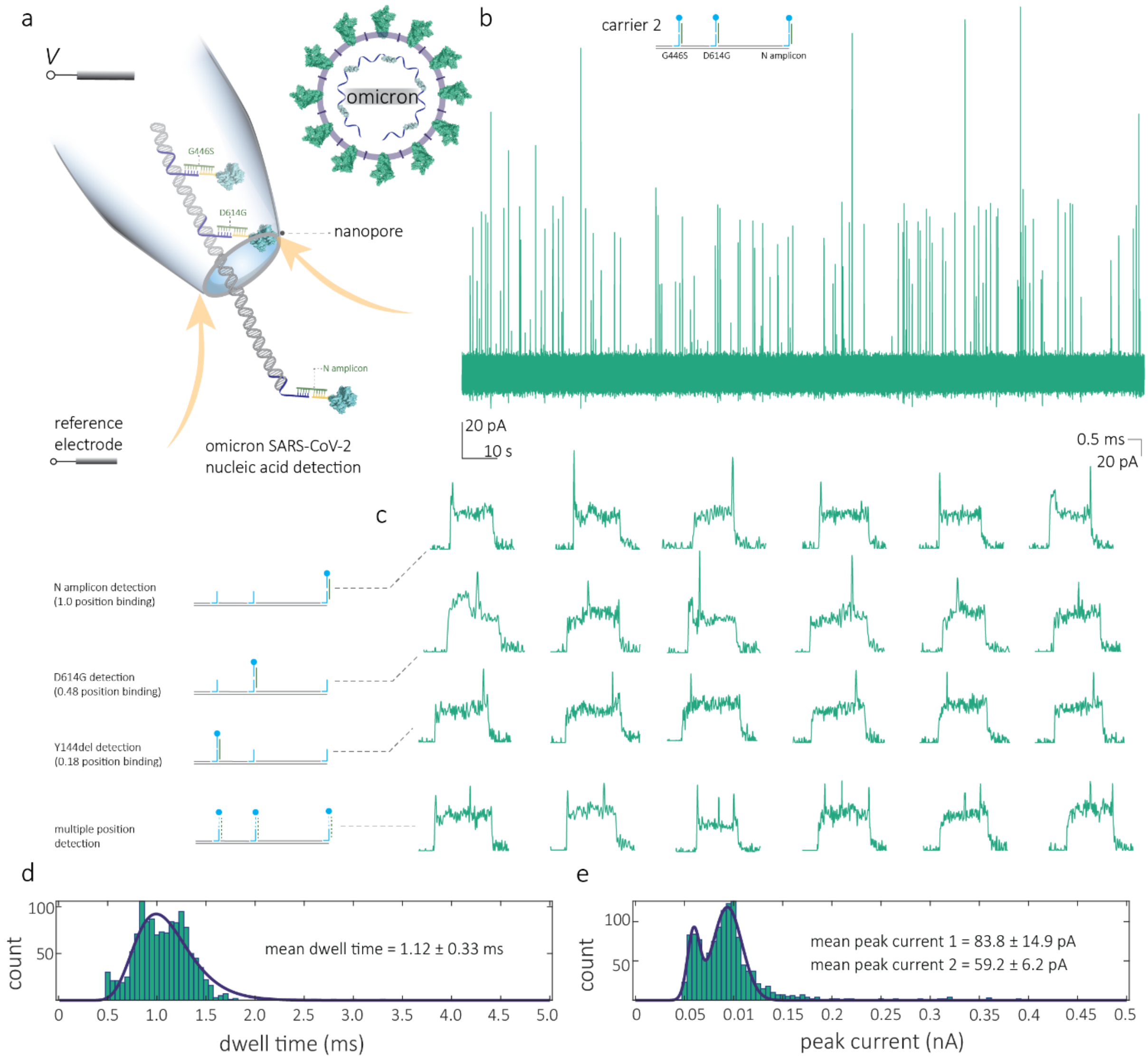

Supplementary Fig. 29 | Test with Omicron variant of SARS-CoV-2.

(a) Schematic showing the translocation of molecular probe (Probe 2) in the presence of Omicron variant of SARS-CoV-2 RNA amplicons. **(b)** Representative current-time trace for the translocation of Probe 2 with Omicron variant of SARS-CoV-2 RNA amplicons (with the region of G446S, D614G, and N gene). **(c)** Zoom-in view of typical translocation events. As for Omicron variant, sub-peaks at all three positions of 0.18, 0.48 and 1 can be detected, implying the presence of G446S, D614G and N gene, respectively. Histograms of translocation dwell time and peak current were summarised in **(d)** and **(e)** with Gaussian function fitting. All the translocation experiments were performed using 200 pM molecular probes in 2 M LiCl buffer (5 mM $MgCl_2$, 10 mM Tris-HCl, 1 mM EDTA, pH = 8) at an applied potential bias of 300 mV.

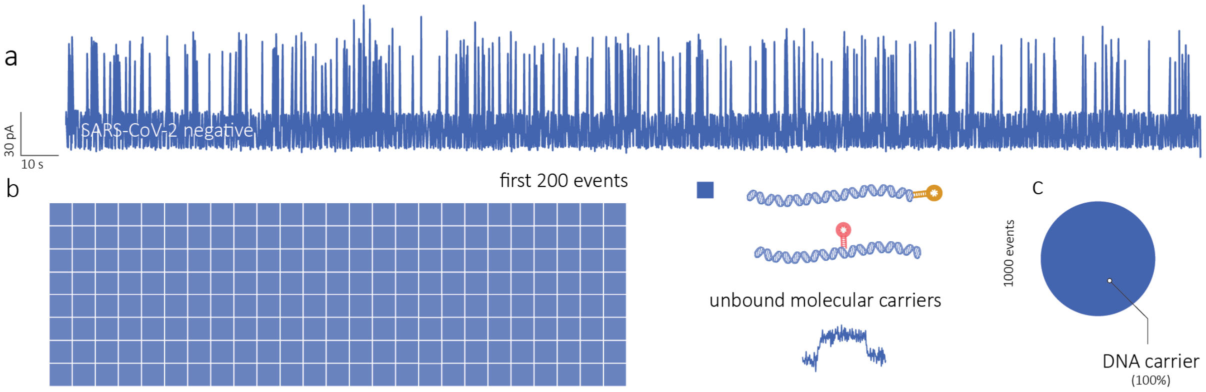

Supplementary Fig. 30 | **Detection of SBA and NBA-modified probes in saliva**

a. Representative current-time traces for SBA and NBA modified molecular probes (200 pM each) in pooled human saliva (>3 people). **b**. The first 200 events are shown in a colour-coded pixel grid, with blue events representing the signal for the molecular probes. Pie charts in **c** show the breakdown of a total of 1,000 events. The translocation experiments were performed in a 1:20 human saliva spiked in 2 M LiCl buffer (5 mM $MgCl_2$, 10 mM Tris-HCl, 1 mM EDTA, pH = 8) with 200 pM of each molecular probe at an applied potential bias of 300 mV.

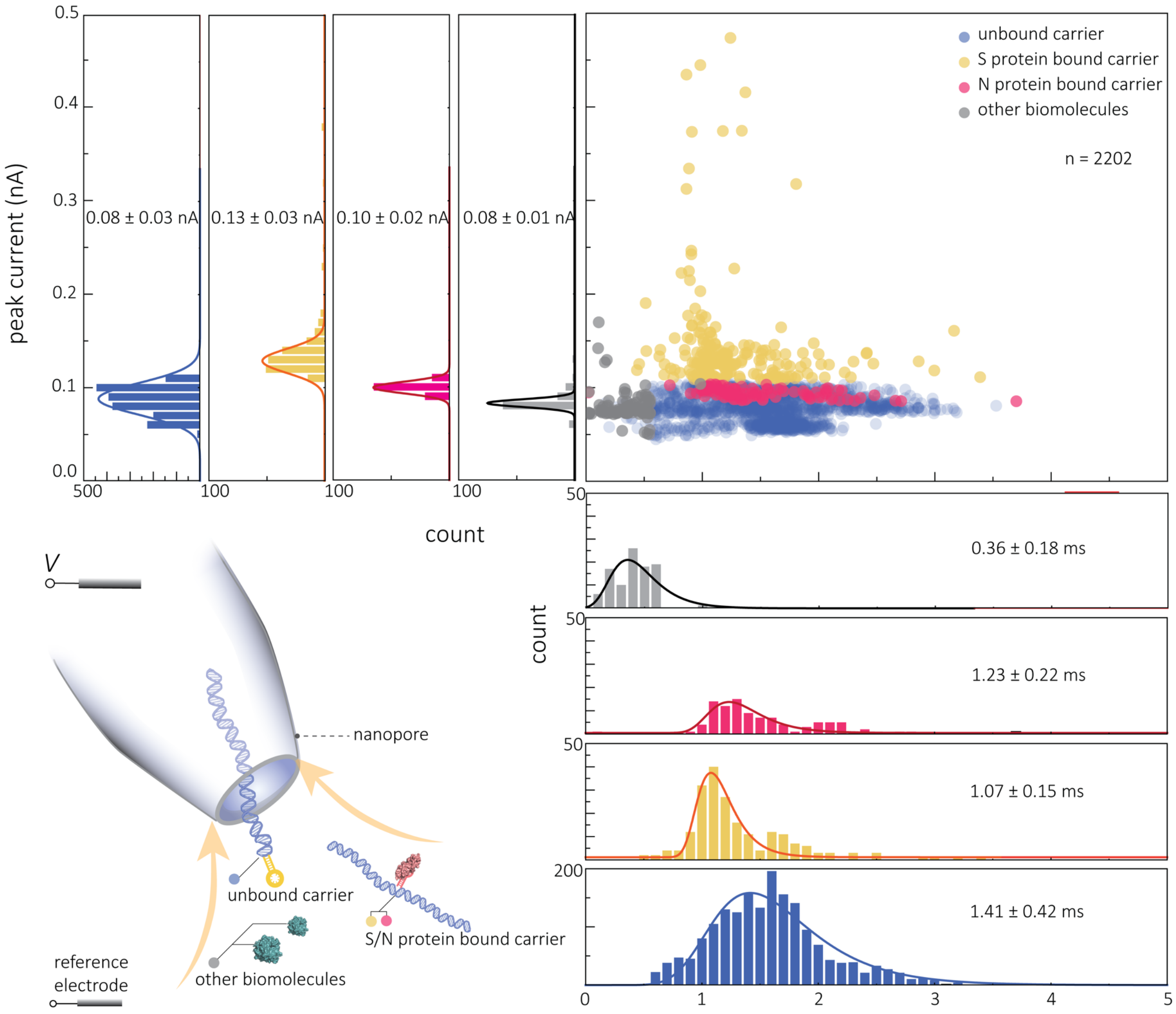

Supplementary Fig. 31 | Statistics for multiplexed detection of S and N protein in saliva.

Scatter plots of peak current versus dwell time for the unbound probe (blue), S protein bound probe (yellow), N protein bound probe (red) and other biomolecules (grey), respectively. The histograms for peak currents are shown on the left, and for dwell time are shown on the bottom. The translocation experiments were performed using 200 pM of each molecular probe in 2 M LiCl buffer (5 mM $MgCl_2$, 10 mM Tris-HCl, 1 mM EDTA, pH = 8) at an applied potential bias of 300 mV.

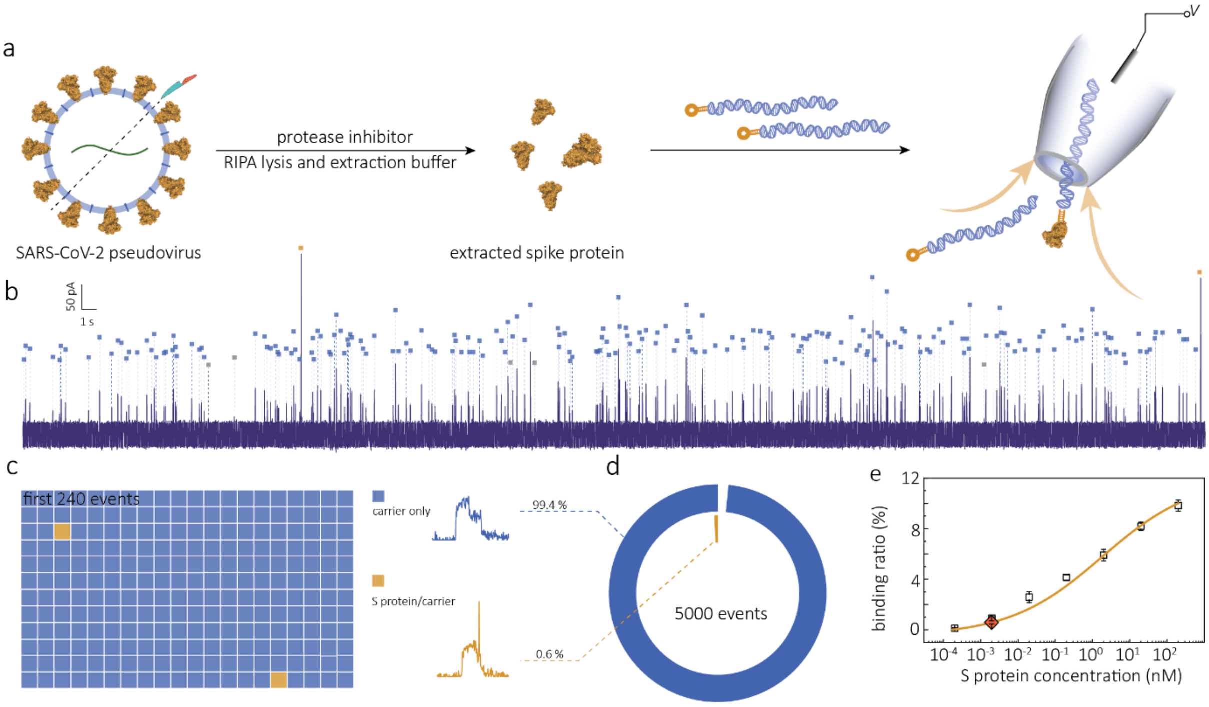

Supplementary Fig. 32 | Digitally multiplexed sensing of pseudovirus and full-length RNA of SARS-CoV-2.

Sensing of pseudovirus S protein. **a**. Schematic showing the workflow of pseudovirus detection. The pseudovirus is firstly lysed by the addition of protease inhibitor, RIPA, and extraction buffer. The protein is then extracted and incubated with the prepared SBA molecular probe, and nanopore measurements are performed. A representative current-time trace for the detection is shown with the molecular probes marked with blue squares, other biomolecules are marked with grey squares, and S protein binding events are marked with yellow squares (**b**). The classification of the first 240 detected probes is shown in a colour-coded pixel grid (**c**), and the breakdown of a total of 5,000 detected probes is shown in (**d**). **e**. The concentration of S protein is estimated according to the calibration curve obtained from **Fig. 2d**.

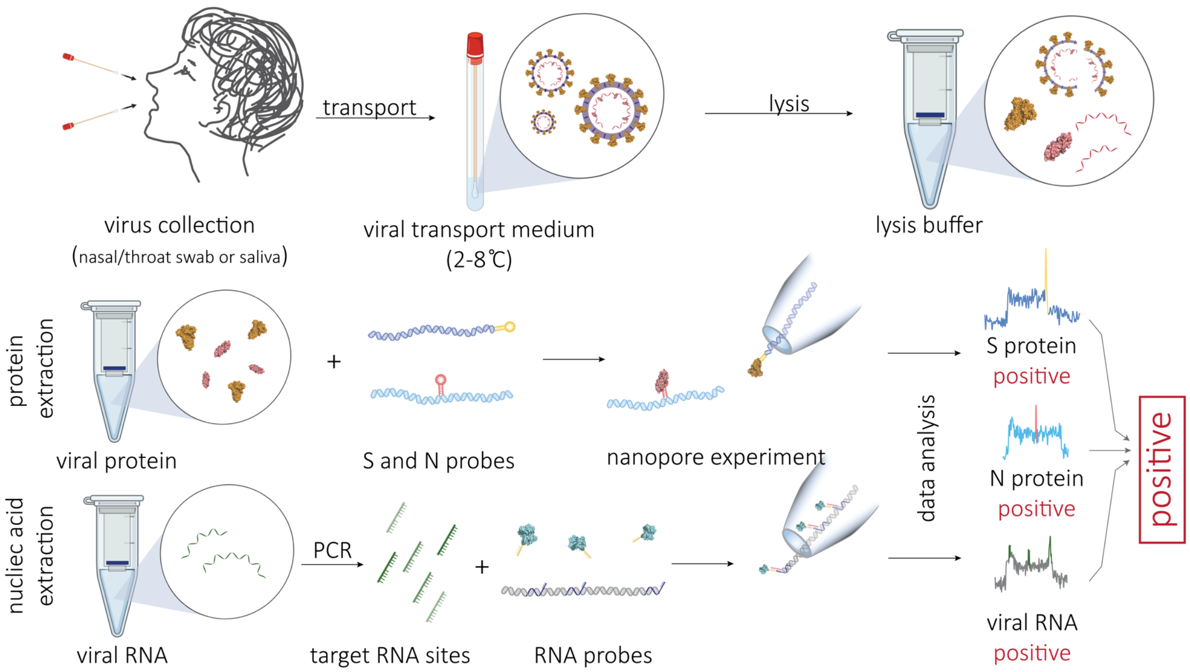

Supplementary Fig. 33 | Workflow of multiplexed detection of antigens and RNAs from patients.

Schematic showing the workflow for simultaneous detection of S protein, N protein, and multiple RNA regions. The samples (nasal swab) collected from patients is lysed to release all the proteins and viral RNA. The lysate is then divided into two portions: one is incubated with S and N protein-specific DNA molecular probes. The other one is incubated with RNA region-specific (or mutation-specific) DNA molecular probes. Both samples are measured in nanopore experiments.

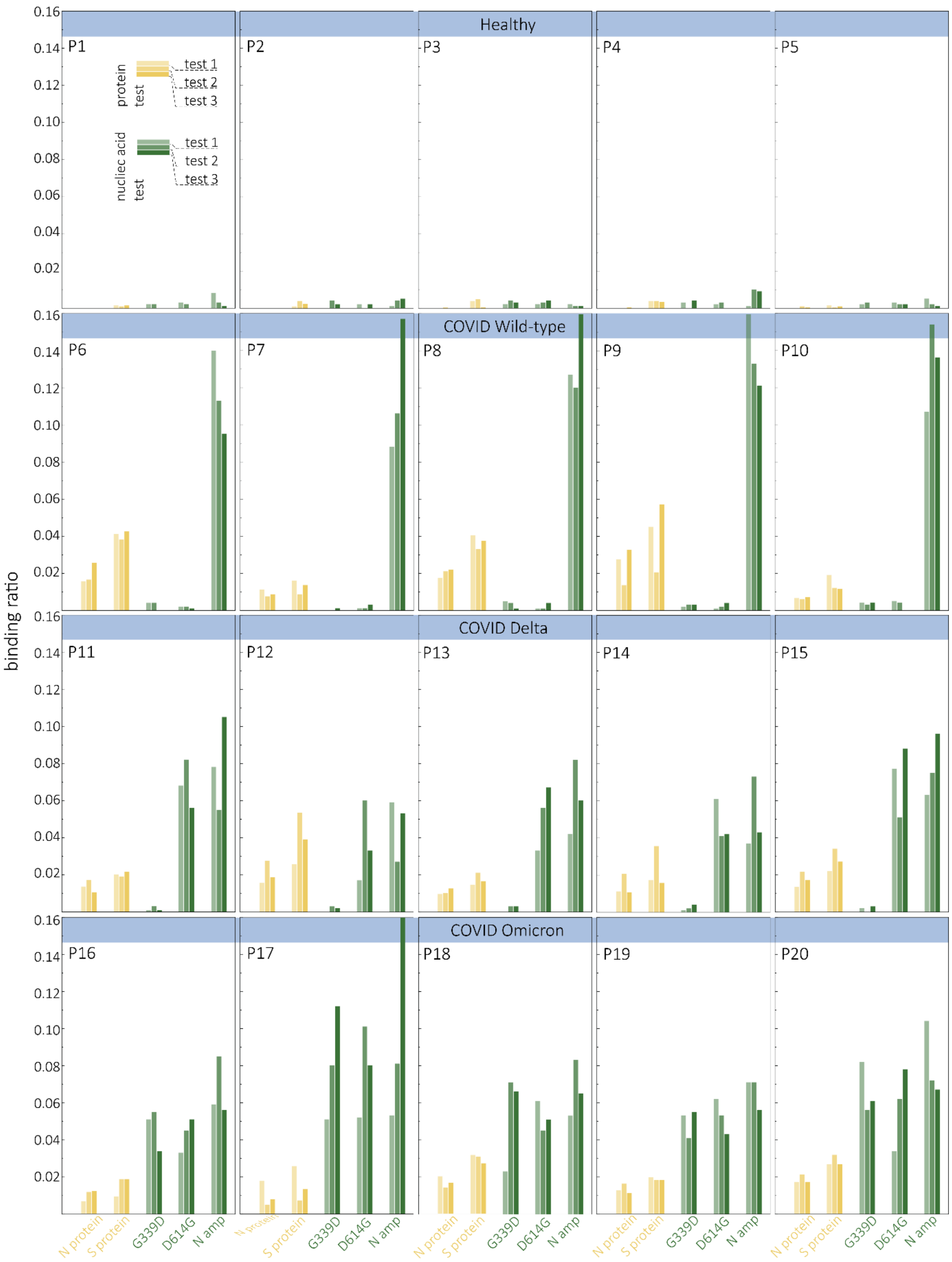

Supplementary Fig. 34 | Statistics for multiplexed detection results of antigens and RNAs from patients.

A summary of the binding ratio for each sub-peak at the respective fractional positions for both protein probes and DNA probes using patient samples (Healthy: P1-P5, COVID wild-type P6-P10, Delta P11-P15, Omicron P16-P20). Each patient sample has been tested three times. The binding ratio was counted by

events with subpeaks based on their fractional positions: for protein detection, N protein 0.35-0.65, S protein 0.85-1.0; for nucleic acid detection, G339D 0.15-0.35, D614G 0.35-0.65, N amplicon 0.85-1.0 over whole events.

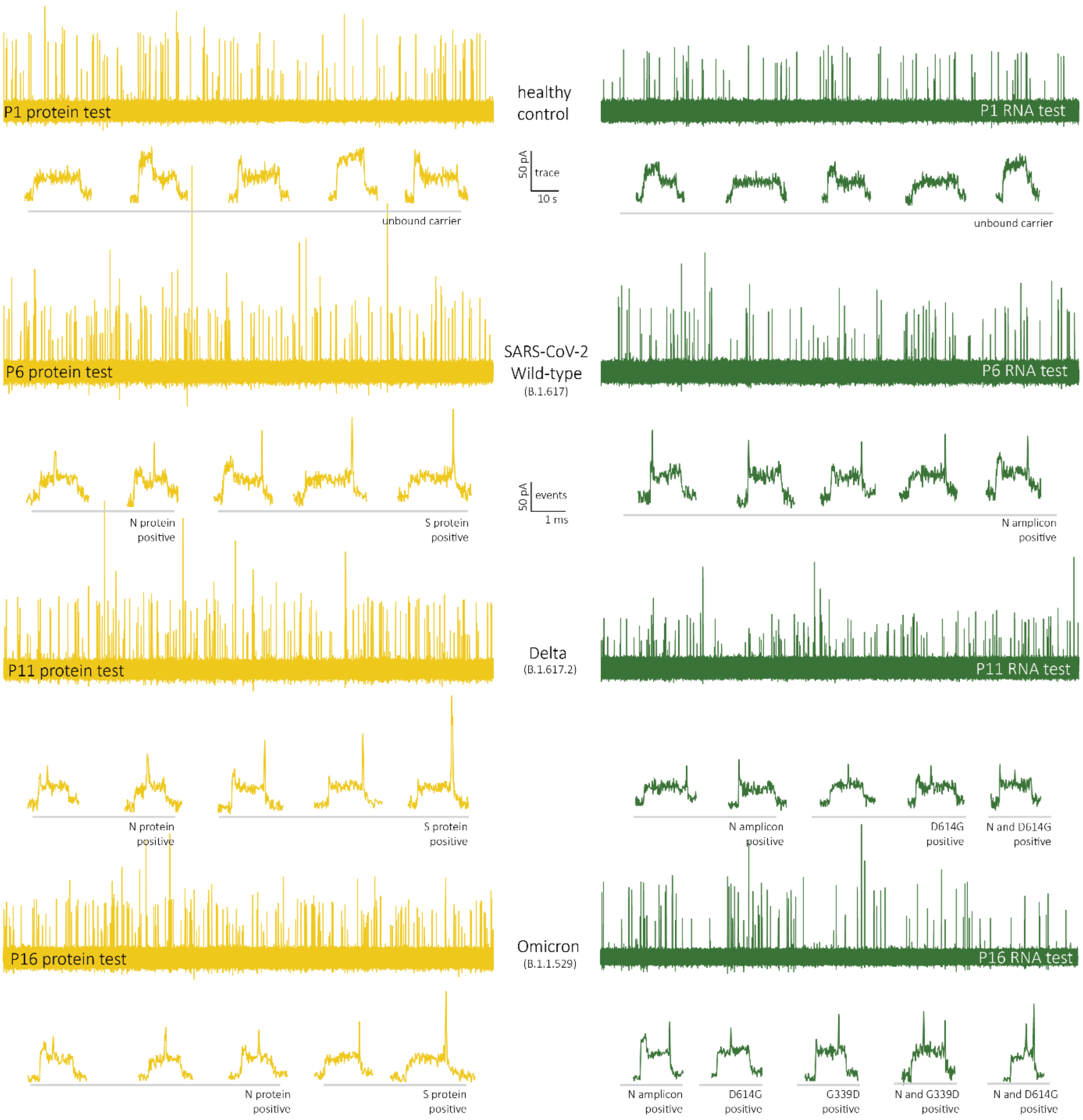

Supplementary Fig. 35 | Representative trace and events for patient sample.

Example I-t traces along with example events for protein and RNA detection of patient samples including P1 (healthy control), P6 (SARS-CoV-2 wild-type), P11 (Delta), and P16 (Omicron).

Supplementary Note 1

Single-molecule events were analysed using the Nanopore App v7.17, a Matlab-based software by Prof. Joshua Edel. A copy of the Nanopore App can be requested from joshua.edel@imperial.ac.uk. This application offers a comprehensive analysis of diverse types of single-molecule data traces. It facilitates tasks such as importing traces, resampling/refiltering, event identification, and exporting all relevant information. The exported data encompasses dwell time, peak current, peak area, subpeak position, subpeak details (time, current), capture rate, power spectral density (PSD), and more. Below summarises our workflow key features, from importing raw data to performing statistical analysis.

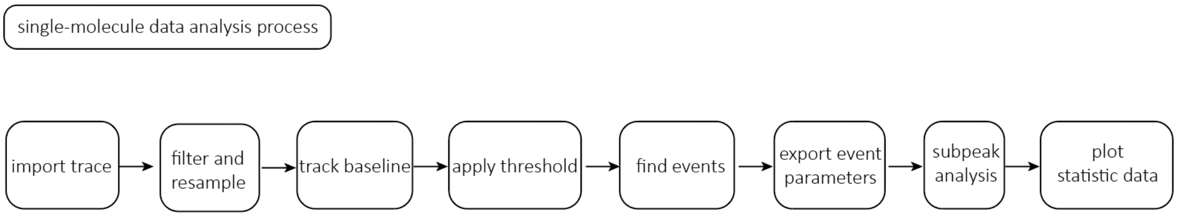

Step 1. Import trace

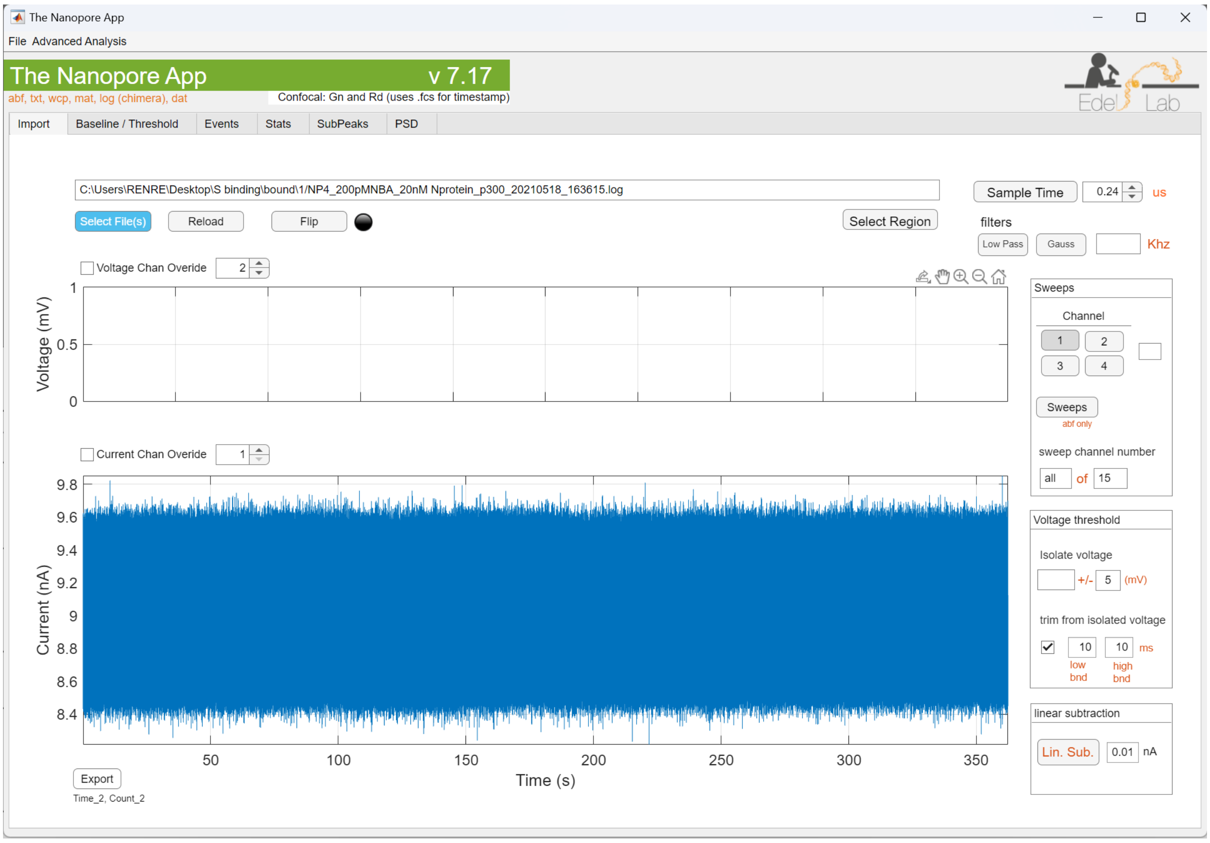

The Nanopore App is compatible with various file formats, including abf (Molecular Devices), wcp (WinWCP), log, mat (Chimera Instruments), dat (Elements), and more. In the figure, we have loaded a segment of raw data pertaining to the detection of the S protein. This example data was digitised using a Chimera VC100 at a sampling rate of 4.16 Mhz.

Step 2. Resample and filter trace

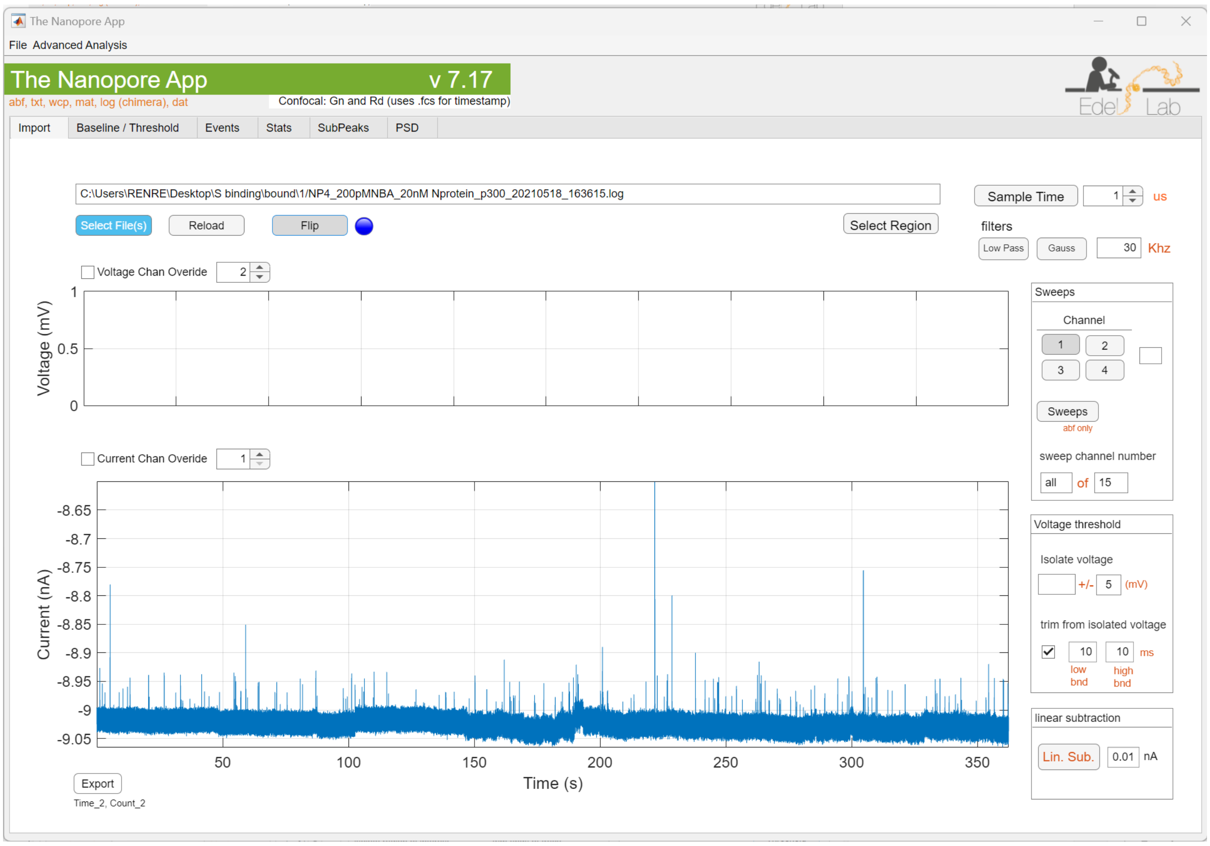

In order to enhance the clarity of the events, it is possible to perform resampling and refiltering. In this particular example, the trace was resampled at 1 µs and subjected to a low pass filter with a cutoff frequency of 30 kHz.

Step 3. Baseline tracking

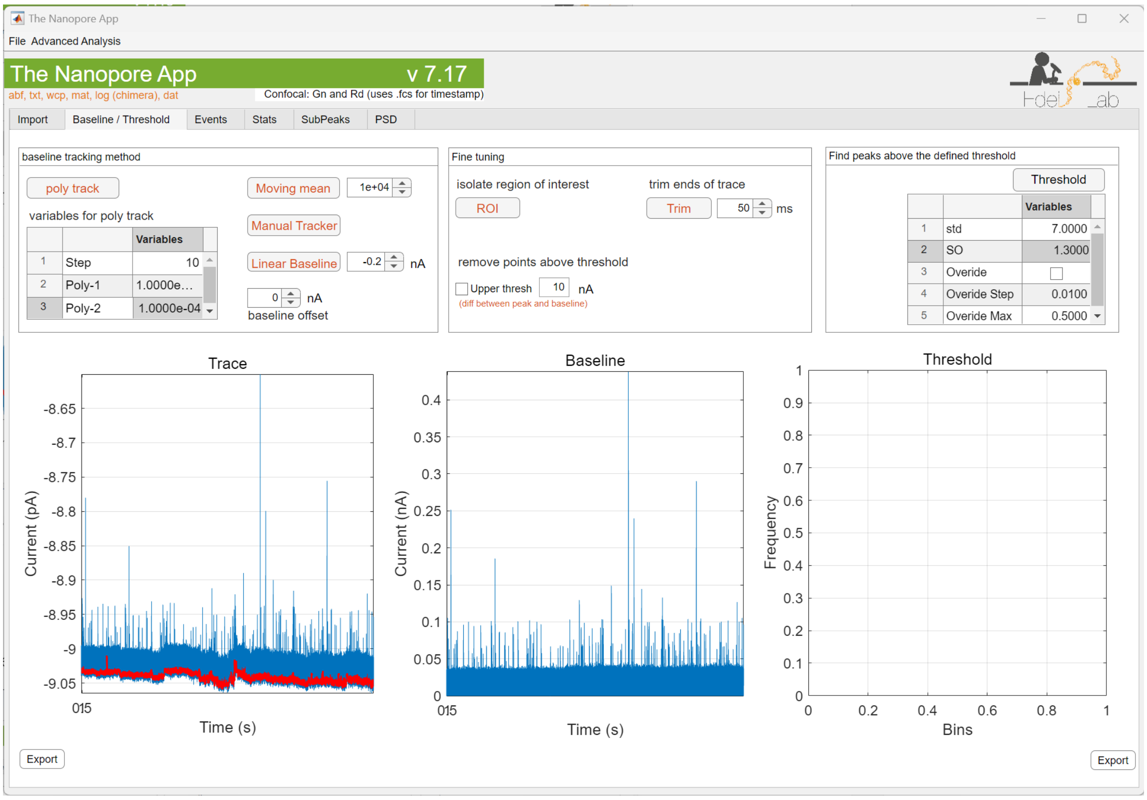

To compensate for signal fluctuations and improve event classification, the baseline was tracked. This involved applying a polynomial fit while adjusting parameters such as 'Step', 'Poly-1', and 'Poly-2'. The 'Step' value determines the sample numbers used in fitting the baseline, while 'Poly-1' and 'Poly-2' are parameters in the polynomial fit.

Typically, the 'Step' number falls within the range of 1-100, depending on the sampling rate of the trace. Higher sampling rate traces can accommodate larger 'Step' values, while lower sampling rate traces require smaller 'Step' values. The chosen 'Step' value also affects the processing speed. By reducing 'Poly-1' and increasing 'Poly-2', the polynomial fit can better capture the baseline fluctuations observed in the raw trace. However, this adjustment may also increase the likelihood of including wide signals in the baseline level. It is crucial to find an appropriate balance for 'Poly-1' and 'Poly-2' to achieve accurate baseline fitting. In this context, we present three examples: two poor fits and one good fit. The first fit

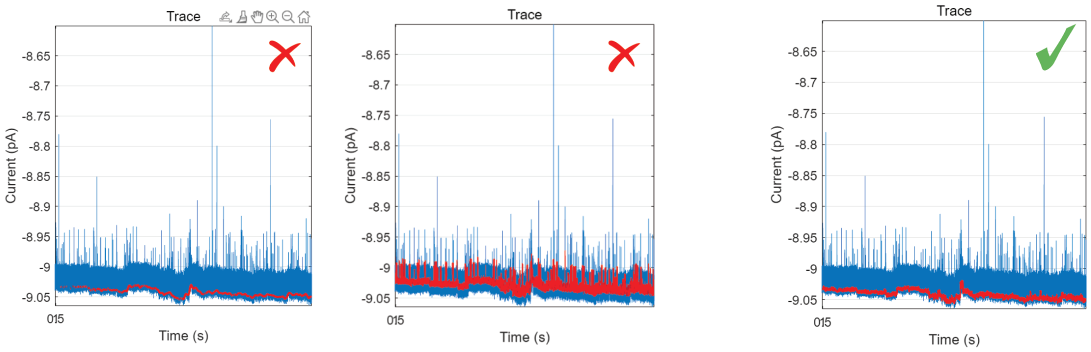

Step 4. Threshold for event classification

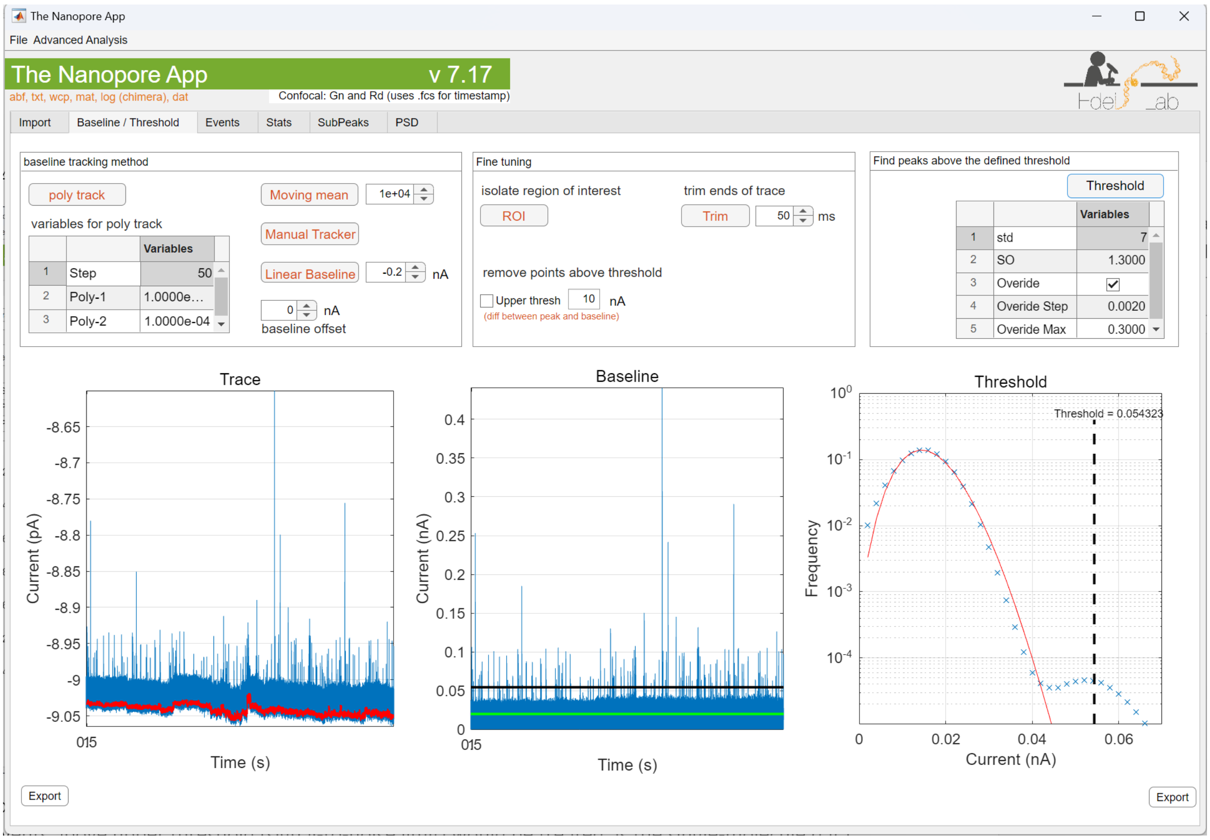

To identify the peaks, an upper threshold (represented by the black line) and a lower threshold (represented by the green line) were applied. This was determined by fitting a Poisson distribution to the open-pore current. In our data sets, typically, a threshold of 7 standard deviations above the mean open-pore current was used to identify events. The peaks of interest were determined based on their position relative to these thresholds. Specifically, any signals surpassing the upper threshold were classified as an event, while the lower threshold determined each event's start and end points.

Step 5. Find events

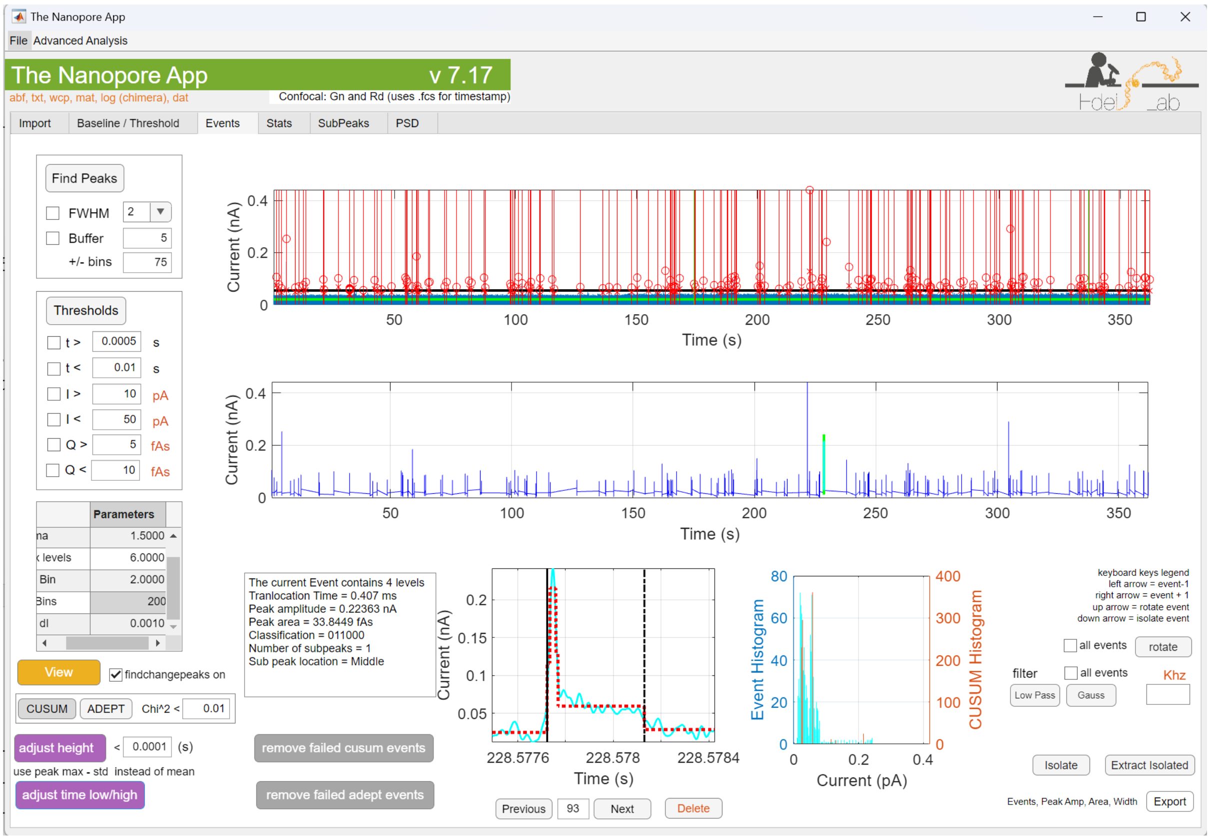

To identify the peaks, the events exceeding the upper threshold can be selected by clicking the 'Find Peaks' button. When the Full-width of half maximum (FWHM) option is chosen, the event's start and end points are determined based on the half of the maximum point of the peak. If FWHM is not selected, the start and end points of the event are determined by the green line (lower threshold) from the previous step. The onset and endpoints are refined using CUSUM (cumulative sums algorithm) fit.[3, 4] For each event, a CUSUM fit (represented by the red dashed line) is employed: Different molecules, such as DNA, protein, peptides, cause different disruptions in the current. The resulting signal has "steps" corresponding to these different molecules' size and charge. The CUSUM algorithm is used to detect these "steps". Once these significant changes or steps are detected, the continuous signal can be segmented into "events", each event corresponding to a particular DNA, protein or DNA/protein-bound complex. This algorithm is based on an abrupt change detection that provides fitting of current blockages, allowing to quickly identify the different levels and subpeaks in each event. Information such as translocation time, peak amplitude, peak area, and subpeak information can be obtained. Additionally, all the information for the identified peaks can be exported by clicking the button located at the bottom-right corner.

Thresholds such as dwell time, peak current, and peak area can be applied. This helps filter out noisy or irrelevant events, ensuring better event selection.

Step 6. Export event parameters

For further analysis, information such as peak amplitude, area, and width can be exported to the Matlab workspace. The exported data can be plotted using various software tools such as Excel, Matlab, or OriginLab.

Step 7. Subpeak analysis

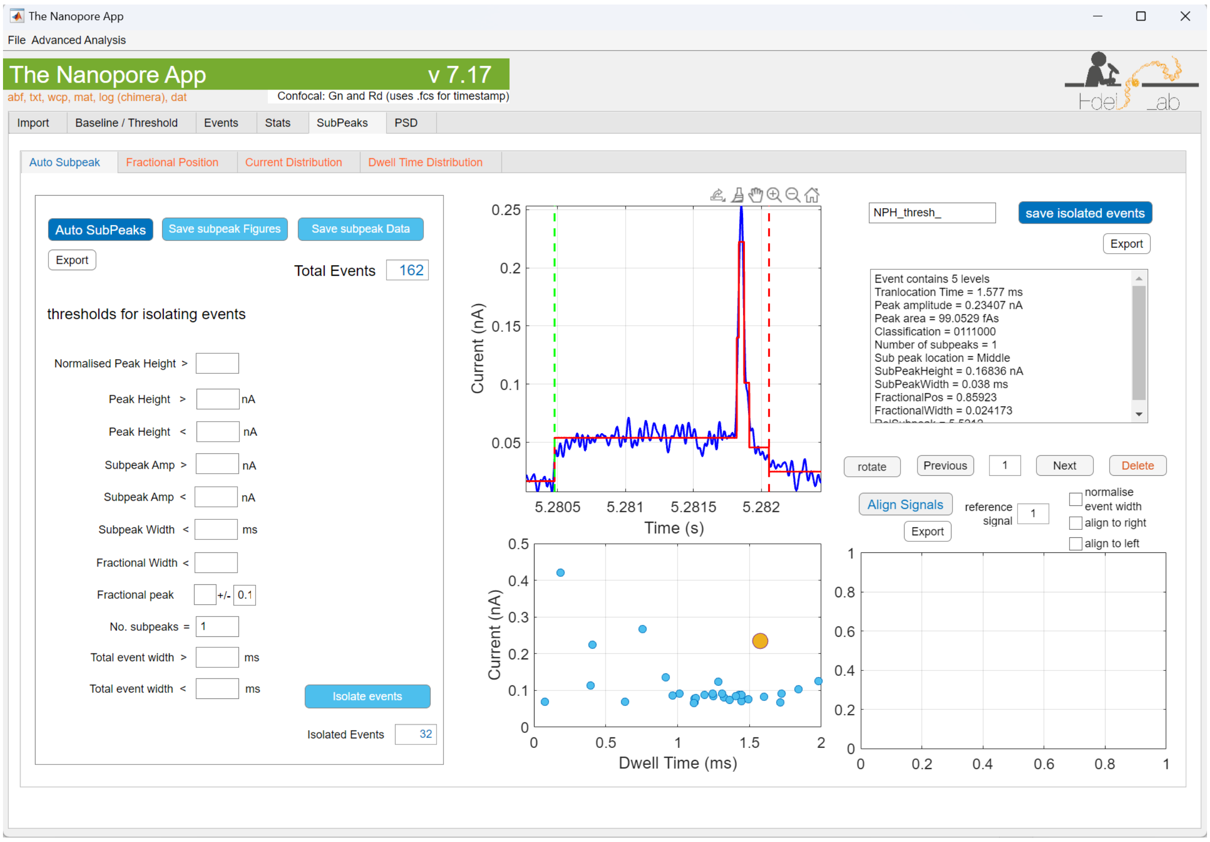

The last step of the data analysis involves extracting the subpeak information. By clicking the 'Auto Subpeaks' button, relevant information such as subpeak amplitude, subpeak dwell time, fractional position of the subpeak, fractional width of the subpeak, number of subpeaks, location of subpeaks could be extracted directly from the CUSUM fits, which has been illustrated in Step 5. Thresholds could be employed to distinguish between genuine positive events and potential false positives due to DNA folding. The fractional position is often used to isolate events associated with subpeaks originating at a defined location. As the subpeak width for the protein is typically smaller than that of folded DNA this can also be used as a threshold. Peak amplitude can also be used as a discriminator (**Supplementary Fig. 36**).

For S protein detection, the threshold for the normalised subpeak position (The 'Fractional Peak' in the Nanopore App interface) has been set to 0.1 ± 0.1 and 0.9 ± 0.1 as the S protein would bound to the SBA located at the end of the molecular carrier. To discriminate the S protein signal and partially folded signal, which is observed at the events' beginning or end, another two thresholds, subpeak width and subpeak/DNA ratio, have been applied. For partial DNA folding, the subpeak always shows a wide rectangular shape with a longer dwell time (0.45 ± 0.23 ms) and is very unlikely to show a narrow, sharp spike due to the limitation with the persistence length (> 50 nm). However, the S protein binding results in a short dwell time and high current amplitude secondary peak due to the relatively larger size of the

S1 protein compared to the folded DNA. Here, the threshold for subpeak width was set to <0.2 ms to select the events with narrow subpeaks where the threshold for subpeak/DNA ratio was set to >1.5 as the folded DNA subpeak/unfolded DNA level was 0.662 ± 0.027 (**Supplementary Fig. 36**). It should be noted that when the translocation event exhibited both folding and protein binding signals, these events were individually examined to verify that all binding events were classified correctly. The representative DNA folded events that can be filtered out, the S protein bound events that can be selected automatically, and the S protein bound events that contain DNA folding, which requires a manual check, were listed in **Supplementary Fig. 37**.

a

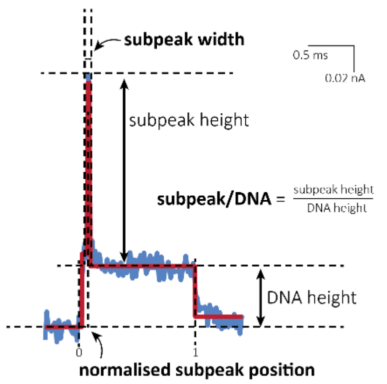

b

	positive event conformation	normalised subpeak position	subpeak width	subpeak/DNA ratio
S protein		0.1 ± 0.1<br>0.9 ± 0.1	< 0.2 ms	> 1.5
N protein		0.5 ± 0.2	< 0.3 ms	n/a
RNA (1.0 site)		0.1 ± 0.1<br>0.9 ± 0.1	< 0.2 ms	n/a
RNA (0.5 site)		0.5 ± 0.2	< 0.2 ms	n/a
RNA (0.2 site)		0.2 ± 0.1<br>0.8 ± 0.1	< 0.2 ms	n/a

Supplementary Fig. 36 | The parameters used for identifying positive subpeak.

a. Schematic of key parameters used for the selection of positive events. b. The threshold value for each parameter in the selection of different signals.

The threshold for the normalised subpeak position for N protein detection has been set to 0.5 ± 0.2. This is in good agreement with the placement of N protein binding site, which is designed to have a fraction position of 0.48 based on the sequence on the molecular carrier. Subpeak amplitude was not used to discriminate between events due to the similarity in amplitude between folded and protein-bound events. The observation of DNA knots in the middle of the translocation event is uncommon (< 0.1 %), and hence, all protein-bound events could be isolated based on normalised peak position and subpeak width (<0.3 ms). Similarly, all folded events were cross-checked manually to ensure all binding events were counted as partial folding will lead to a slight shift in the fractional position. For RNA 1.0 site, we use a similar strategy as this is N amplicon site, and it is responsible for confirmation of the positive signal and RNA quantification. Therefore, in addition to applying a 0.1 ± 0.1 and 0.9 ± 0.1 normalised subpeak threshold and <0.2 ms subpeak width threshold, the events were always checked manually to ensure accurate counting.

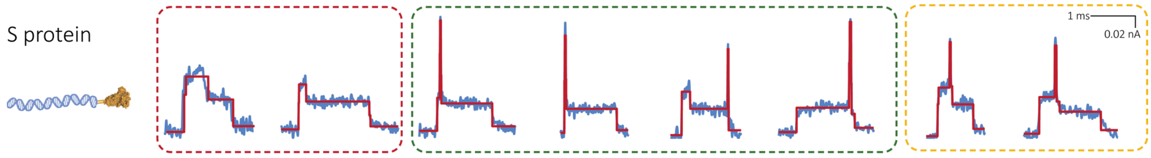

translocation time (ms)	1.0906	1.6224	1.2326	1.186	1.0618	1.296	0.79296	1.0718
peak amplitude (nA)	0.11438	0.10826	0.2353	0.19657	0.15423	0.22517	0.16937	0.18119
number of subpeaks	1	1	1	1	2	1	1	1
subpeak height 1 (nA)	0.035712	0.029813	0.16117	0.13191	0.028185	0.16131	0.10721	0.1228167
subpeak width 1 (ms)	**0.49344**	**0.15936**	**0.01728**	**0.022**	0.18816	**0.03072**	**0.02352**	**0.02688**
fractional position 1	**0.036972**	**0.0059172**	**0.051402**	**0.018613**	0	**0.91259**	**0.33475**	**0.25081**
subpeak 1 to DNA	**0.7114**	**0.613**	**2.0322**	**3.0362**	0.6447	**3.5201**	**1.9575**	**2.7237**
subpeak height 2 (nA)	0	0	0	0	0.098829	0	0	0
subpeak width 2 (ms)	0	0	0	0	**0.0168**	0	0	0
fractional position 2	0	0	0	0	**0.98101**	0	0	0
subpeak 2 to DNA	0	0	0	0	**2.2607**	0	0	0
possible conformation during translocation								

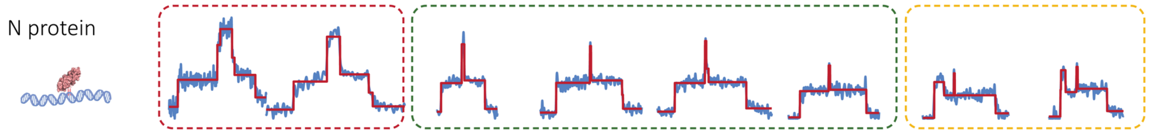

translocation time (ms)	1.7904	1.8493	0.969	1.6282	1.6531	1.4683	1.4011	1.0382
peak amplitude (nA)	0.15285	0.14932	0.13257	0.12483	0.13747	0.091755	0.083551	0.091213
number of subpeaks	1	1	1	1	1	1	2	2
subpeak height 1 (nA)	0.085054	0.072977	0.065098	0.057935	0.068251	0.040475	0.022903	0.030823
subpeak width 1 (ms)	**0.334**	**0.304128**	**0.057**	**0.02688**	**0.0288**	**0.03024**	0.23521	0.09744
fractional position 1	**0.54671**	**0.42193**	**0.47059**	**0.50472**	**0.42044**	**0.43249**	0	0.012945
subpeak 1 to DNA	1.8759	1.5945	1.3563	1.2345	1.388	0.8728	1.5831	0.6686
subpeak height 2 (nA)	0	0	0	0	0	0	0.0378626	0.036311
subpeak width 2 (ms)	0	0	0	0	0	0	**0.01972**	**0.02688**
fractional position 2	0	0	0	0	0	0	**0.32025**	**0.34304**
subpeak 2 to DNA	0	0	0	0	0	0	0.8136	0.7876
possible conformation during translocation								

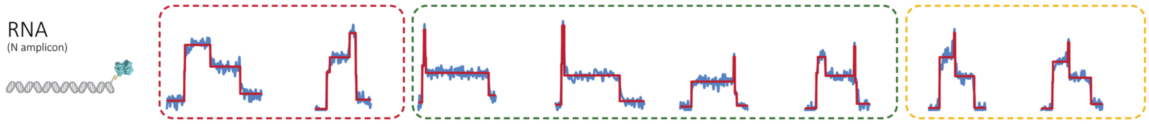

translocation time (ms)	1.2422	0.64176	1.486	1.232	1.0147	0.90384	0.73248	0.86688
peak amplitude (nA)	0.11216	0.14403	0.12207	0.13711	0.086642	0.10127	0.1509	0.115046
number of subpeaks	1	1	1	1	1	2	1	2
subpeak height 1 (nA)	0.033473	0.041594	0.063476	0.082288	0.041381	0.028442	0.087403	0.063139
subpeak width 1 (ms)	**0.56256**	**0.13104**	**0.029**	**0.0444**	**0.02352**	0.168	**0.03024**	**0.03113**
fractional position 1	**0.010819**	**0.76963**	**0.025572**	**0.013221**	**0.93046**	0.037175	**0.46789**	**0.411216**
subpeak 1 to DNA	0.6325	1.4695	1.4593	1.7036	0.9848	1.577	1.6086	1.4159
subpeak height 2 (nA)	0	0	0	0	0	0.057625	0	0
subpeak width 2 (ms)	0	0	0	0	0	**0.02321**	0	0
fractional position 2	0	0	0	0	0	**0.91302**	0	0
subpeak 2 to DNA	0	0	0	0	0	1.1759	0	0
possible conformation during translocation								

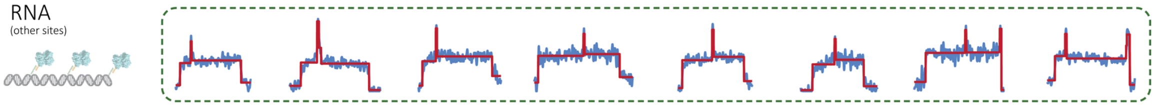

translocation time (ms)	1.353	1.5254	1.531	1.867	1.398	1.1189	2.016	1.265
peak amplitude (nA)	0.088046	0.12344	0.101995	0.082852	0.111225	0.086597	0.090326	0.096095
number of subpeaks	1	1	1	1	1	1	2	1
subpeak height 1 (nA)	0.03206	0.07345	0.062311	0.035593	0.057788	0.033605	0.031637	0.044923
subpeak width 1 (ms)	**0.024**	**0.04032**	**0.027**	**0.026**	**0.035**	**0.02352**	**0.02688**	**0.026**
fractional position 1	**0.17295**	**0.23348**	**0.19268**	**0.5549**	**0.48355**	**0.42943**	**0.50833**	**0.15731**
subpeak 1 to DNA	0.7773	1.6042	1.2426	0.8262	0.9707	0.6896	0.6283	0.9018
subpeak height 2 (nA)	0	0	0	0	0	0	0.02383	0.046661
subpeak width 2 (ms)	0	0	0	0	0	0	**0.02681**	**0.061**
fractional position 2	0	0	0	0	0	0	**0.97312**	**0.90342**
subpeak 2 to DNA	0	0	0	0	0	0	0.6275	0.8898
possible conformation during translocation								

Supplementary Fig. 37 | Example events that been isolated using the thresholds.

Utilising specific thresholds, we can distinguish and separate events with relevant subpeaks. Signals from molecular carriers, regardless of their folded or unfolded state, are excluded (indicated in the red box). Events that satisfy all threshold criteria for subpeak information are identified as potential positive signals (highlighted in the green box). For events exhibiting both folding and protein-binding indications, individual assessments were made to ensure proper classification of all binding instances (shown in the yellow box). The events we listed here are all recorded with different nanopores.

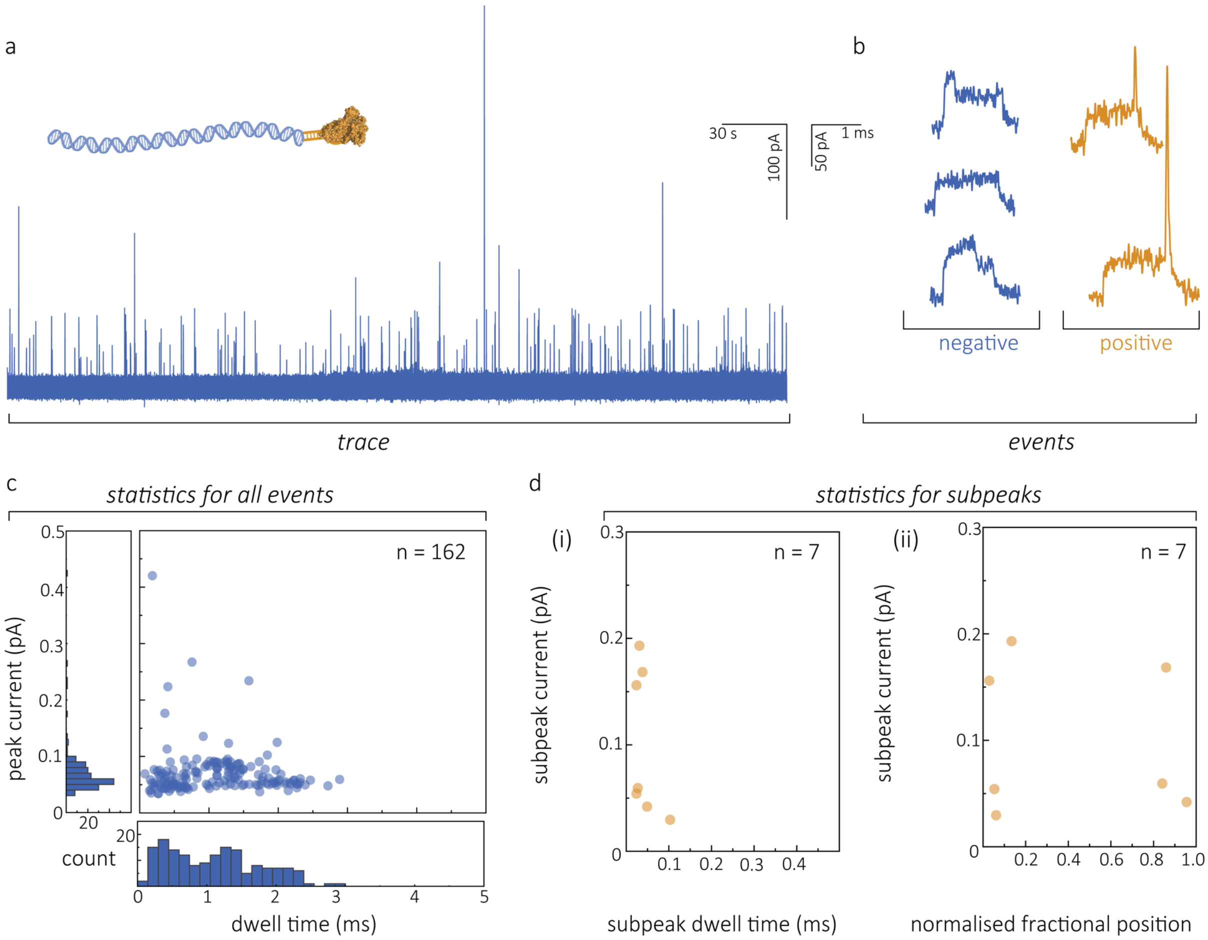

Supplementary Fig. 38 | Exported current-time trace, events and statistics from an example trace.

(a) Partial I-t trace for an S protein-bound molecular probe translocation experiment. **(b)** Representative negative signals and positive signals in the I-t trace. **(c)** The peak current - dwell time scatter plot, histogram of dwell time, and histogram of the peak current of all events in the example trace. **(d)** The extracted subpeak data **(i)** subpeak current – subpeak dwell time and **(ii)** subpeak current – normalised fractional position.

Supplementary Tables

Supplementary Table 1. Aptamer probes for target S and N proteins

Oligos	Sequences (5' to 3')	Specification
**SBA probe**	5'- AGGTCGCCGCCC TTTT TTTT **CACGCATAACGTCTTGCGGGGCGGCGGGTTGAGAGGATGTCGGGTGGTTATGCGTG** -3'	Complementary sequences to DNA carrier are underlined. Aptamer sequences are in bold.
**NBA probe**	5'- TGA GGT TTG GGC GGC GAC CTG GCT CAA GCG AGT GGA AAA AGT TAG AAG CTT AAA AAC TTA CGC AGC TTTT TTTT **GCTGGATGTCGCTTACGACAATATTCCTTAGGGGCACCGCTACATTGACACATCCAGC** -3'	Complementary sequences to DNA carrier are underlined. Aptamer sequences are in bold.

Supplementary Table 2. Sequences for N gene detection

Oligos	Sequences (5' to 3')	Specification
**N gene amplicon**	5'-AGC GAA ATG CA C CCC G CA TTA C GTT TGG TGG ACC CTC AGA TTC AAC TGG CAG TAA CCA GAA TGG AGA ACG CAG TGG GGC GC G ATC AAA ACA A -3'	Sequences in blue are complementary to probe A on DNA carrier. Sequences in green are complementary to probe B.
**Forward primer**	gaaattaatacgactcactataggg AGCGAAATGCACCCCGCATTAC	Sequences underlined are upstream of T7 promoter sequence.
**Reverse primer**	TTGTTTTGATCGCGCCCCACTG	
**N gene probe A**	5'-Phos- GGG CGG CGA CCT TTT TTT **CCA CTG CGT TCT CCA TTC TGG TTA CTG CCA** - 3'	Probe to target first half of target RNA. Sequences underlined are complementary to DNA carrier. Sequences in bold are probe to target.
**N gene probe B**	5'- **GTT GAA TCT GAG GGT CCA CCA AAC GTA ATG** TTT TTT - biotin-3'	Probe to target second half of target RNA.

Supplementary Table 3. Sequences for S gene detection

Oligos	Sequences (5' to 3')	Specification
**S gene amplicon**	5'- ATG TCC TTC CCT CAG TCA G CAC CTC ATG GTG TAG TCT TCT TGC ATG TGA CTT ATG TCC CTG CAC AAG AAA AGA ACT T CA C AAC T GC TCC TGC CAT TTG T -3'	Sequences in blue are complementary to probe A on DNA carrier. Sequences in green are complementary to probe B.
**Forward primer**	gaaattaatacgactcactataggg ATGTCCTTCCCTCAGTCAGCAC	Sequences underlined are upstream of T7 promoter sequence.
**Reverse primer**	ACAAATGGCAGGAGCAGTTGTG	
**S gene probe A**	5'-Phos- TGC GCC TTC TCC CTG TAC CTG AAT CAA TGT TAG GTT TTT TTT **TGA AGT TCT TTT CTT GTG CAG GGA CAT AAG** - 3'	Probe to target first half of target RNA. Sequences underlined are complementary to DNA carrier. Sequences in bold are probe to target.
**S gene probe B**	5'- **TCA CAT GCA AGA AGA CTA CAC CAT GAG GTG** TTT TTT -biotin-3'	Probe to target second half of target RNA.

Supplementary Table 4. Sequences for ORF1b gene detection

Oligos	Sequences (5' to 3')	Specification
**ORF1b gene amplicon**	5’- CCC TGT GGG TTT TAC ACT TAA AAA C AC AGT CTG TAC CGT CTG CGG TAT GTG GAA AGG TTA TGG CTG TAG TTG TGA TCA ACT CCG CGA ACC CAT GC T TCA G TC AGC TGA TGC ACA ATC GT -3’	Sequences in blue are complementary to probe A on DNA carrier. Sequences in green are complementary to probe B.
**Forward primer**	gaaattaatacgactcactataggg CCC TGT GGG TTT TAC ACT TAA	Sequences underlined are upstream of T7 promoter sequence.
**Reverse primer**	ACG ATT GTG CAT CAG CTG A	
**ORF1b gene probe A**	5’-Phos- CCA TCT CGC TTT CCA CTC CAG AGC CAG TCT CG TTT TTT **GGT TCG CGG AGT TGA TCA CAA CTA CAG CCA** - 3'	Probe to target first half of target RNA. Sequences underlined are complementary to DNA carrier. Sequences in bold are probe to target.
**ORF1b gene probe B**	5'- **TAA CCT TTC CAC ATA CCG CAG ACG GTA CAG** TTT TTT - biotin-3'	Probe to target second half of target RNA.

Supplementary Table 5. Sequences for D614G mutation detection

Oligos	Sequences (5' to 3')	Specification
**D614G gene amplicon**	5'- TGT TCT TTT GGT GGT GTC AGT G TT ATA ACA CCA GGA ACA AAT ACT TCT AAC CAG GTT GCT GTT CTT TAT CAG GGT GTT AAC TGC ACA GA A GTC CCT GTT GCT ATT CAT GCA -3'	Sequences in blue are complementary to probe A on DNA carrier. Sequences in green are complementary to probe B. Mutation bases are highlighted in red.
**Forward primer**	gaaattaatacgactcactataggg TGTTCTTTTGGTGGTGTCAGTG	Sequences underlined are upstream of T7 promoter sequence.
**Reverse primer**	TGCATGAATAGCAACAGGGACT	
**D614G gene probe A (9-base)**	5'-Phos- TGC GCC TTC TCC CTG TAC CTG AAT CAA TGT TAG GTT AAA AAA **AAC ACC CTG** - 3'	Probe to target first half of target RNA. Sequences underlined are complementary to DNA carrier. Sequences in bold are designed to probe the mutation target.
**D614G gene probe A (13-base)**	5'-Phos- TGC GCC TTC TCC CTG TAC CTG AAT CAA TGT TAG GTT AAA AAA **TTA ACA CCC TGA T** - 3'	
**D614G gene probe A (17-base)**	5'-Phos- TGC GCC TTC TCC CTG TAC CTG AAT CAA TGT TAG GTT AAA AAA **AGT TAA CAC CCT GAT AA** - 3'	
**D614G gene probe A (21-base)**	5'-Phos- TGC GCC TTC TCC CTG TAC CTG AAT CAA TGT TAG GTT AAA AAA **GCA GTT AAC ACC CTG ATA AAG** - 3'	
**D614G gene probe B**	5'- **AAC AGC AAC CTG GTT AGA AGT ATT TGT TCC** TTT TTT - biotin-3'	Probe to target second half of target RNA.

Supplementary Table 6. Sequences for Y144del mutation detection

Oligos	Sequences (5' to 3')	Specification
**Y144del gene amplicon**	5'- ACG CTA CTA ATG TTG TTA TTA AAG T CT G TG AAT TTC AAT TTT GTA ATG ATC CAT TTT T GGG TGT TT A TTA CCA CAA AA ACA ACA AAA GTT GGA TGG AAA GTG AGT TCA GAG T -3'	Sequences in blue are complementary to probe A on DNA carrier. Sequences in green are complementary to probe B. Mutation bases (delete) are highlighted in red.
**Forward primer**	gaaattaatacgactcactataggg ACGCTACTAATGTTGTTATTAAAGT	Sequences underlined are upstream of T7 promoter sequence.
**Reverse primer**	ACTCTGAACTCACTTTCCATCC	
**Y144del gene probe A**	5'-Phos- CCA TCT CGC TTT CCA CTC CAG AGC CAG TCT CG AAA AAA **TTG TGG TAA ACA CCC** - 3'	Probe to target first half of target RNA. Sequences underlined are complementary to DNA carrier. Sequences in bold are probed to target.
**Y144del gene probe B**	5'- **AAA AAT GGA TCA TTA CAA AAT TGA AAT TCA** TTT TTT - biotin-3'	Probe to target second half of target RNA.

Supplementary Table 7. Sequences for G446S mutation detection

Oligos	Sequences (5' to 3')	Specification
**G446S gene amplicon**	5'- CAG ATG ATT TTA CAG GCT GCG TTA TAG CTT GGA ATT CTA ACA ATC TTG ATT CT A AGG TTA GTG GTA ATT ATA ATT ACC TGT ATA GAT TGT TTA GGA AGT CTA ATC TCA AAC CTT TTG -3'	Sequences in blue are complementary to probe A on DNA carrier. Sequences in green are complementary to probe B. Mutation bases are highlighted in red.
**Forward primer**	gaaattaatacgactcactataggg CAGATGATTTTACAGGCTGCGT	Sequences underlined are upstream of T7 promoter sequence.
**Reverse primer**	CAAAAGGTTTGAGATTAGACTTCCT	
**G446S gene probe A**	5'-Phos- CCA TCT CGC TTT CCA CTC CAG AGC CAG TCT CG AAA AAA **TAC CAC TAA CCT T**- 3'	Probe to target first half of target RNA. Sequences underlined are complementary to DNA carrier. Sequences in bold are probed to target.
**G446S gene probe B**	5'- **AGA ATC AAG ATT GTT AGA ATT CCA AG** TTT TTT - biotin-3'	Probe to target second half of target RNA.

Supplementary Table 8. Sequences for G339D mutation detection

Oligos	Sequences (5' to 3')	Specification
**G339D gene amplicon**	5'- AAC TTT AGA GTC CAA CCA ACA GAA TCT ATT G TT AGA TTT CCT AAT ATT ACA AAC TTG TGC C CT TTT GAT GAA GT T TTT AAC GCC ACC AGA TTT GCA TCT -3'	Sequences in blue are complementary to probe A on DNA carrier. Sequences in green are complementary to probe B. Mutation bases are highlighted in red.
**Forward primer**	gaaattaatacgactcactataggg AACTTTAGAGTCCAACCAACAGAA	Sequences underlined are upstream of T7 promoter sequence.
**Reverse primer**	AGATGCAAATCTGGTGGCGT	
**G339D gene probe A**	5'-Phos- CCA TCT CGC TTT CCA CTC CAG AGC CAG TCT CG TTT TTT **ACT TCA T CA AAA G**- 3'	Probe to target first half of target RNA. Sequences underlined are complementary to DNA carrier. Sequences in bold are probed to target.
**G339D gene probe B**	5'- **GGC ACA AGT TTG TAA TAT TAG GAA ATC TAA** TTT TTT -biotin-3'	Probe to target second half of target RNA.

Supplementary Table 9. RT-qPCR data for the Delta and Omicron patient samples.

Samples	CT Mean	Date	Binding ratio
delta 1	26.68	23.11.21	0.079
delta 2	28.95	08.12.21	0.046
delta 3	27.16	29.11.21	0.061
delta 4	28.99	03.12.21	0.051
delta 5	26.98	07.12.21	0.078
omicron 1	30.79	04.05.22	0.067
omicron 2	26.65	25.04.22	0.099
omicron 3	30.32	22.04.22	0.067
omicron 4	29.92	09.05.22	0.066
omicron 5	29.85	12.05.21	0.081

Supplementary Table 10. P-value between healthy control and patients with COVID.

N protein			
	P6	P11	P16
P1	0.00922	0.00918	0.01474
	**	**	*
S protein			
	P6	P11	P16
P1	0.00058	0.00078	0.02383
	***	***	*
G339D			
	P6	P11	P16
P1	N.A.	N.A.	0.00989
	N.A.	N.A.	**
D614G			
	P6	P11	P16
P1	N.A.	0.00625	0.00822
	N.A.	**	**
N amplicon			
	P6	P11	P16
P1	0.00685	0.01378	0.09974
	**	*	**

Supplementary references

1. Perry D, Momotenko D, Lazenby RA, Kang M, Unwin PR. Characterisation of Nanopipettes. *Anal Chem* **88**, 5523-5530 (2016).

2. Wrapp D*, et al.* Cryo-EM structure of the 2019-nCoV spike in the prefusion conformation. *Science* **367**, 1260-+ (2020).

3. Raillon C, Granjon P, Graf M, Steinbock LJ, Radenovic A. Fast and automatic processing of multi-level events in nanopore translocation experiments. *Nanoscale* **4**, 4916-4924 (2012).

4. Forstater JH*, et al.* MOSAIC: A Modular Single-Molecule Analysis Interface for Decoding Multistate Nanopore Data. *Analytical Chemistry* **88**, 11900-11907 (2016).
