# Supplementary material for: Multiplexed detection of viral antigen and RNA using nanopore sensing and encoded molecular probes
Source: Nat Commun. 2023 Nov 14;14:7362. doi: 10.1038/s41467-023-43004-9 (PMC10646045; doi:10.1038/s41467-023-43004-9)
Supplement: Supplementary file 3 — Reporting Summary [file 41467_2023_43004_MOESM3_ESM.pdf]

nature portfolio

Corresponding author(s): Joshua B. Edel

Last updated by author(s): Oct 3, 2023

# Reporting Summary

Nature Portfolio wishes to improve the reproducibility of the work that we publish. This form provides structure for consistency and transparency in reporting. For further information on Nature Portfolio policies, see our Editorial Policies and the Editorial Policy Checklist.

## Statistics

For all statistical analyses, confirm that the following items are present in the figure legend, table legend, main text, or Methods section.

| n/a | Confirmed | |
|---|---|---|
| ☐ | ☒ | The exact sample size (*n*) for each experimental group/condition, given as a discrete number and unit of measurement |
| ☐ | ☒ | A statement on whether measurements were taken from distinct samples or whether the same sample was measured repeatedly |
| ☐ | ☒ | The statistical test(s) used AND whether they are one- or two-sided<br>*Only common tests should be described solely by name; describe more complex techniques in the Methods section.* |
| ☒ | ☐ | A description of all covariates tested |
| ☒ | ☐ | A description of any assumptions or corrections, such as tests of normality and adjustment for multiple comparisons |
| ☐ | ☒ | A full description of the statistical parameters including central tendency (e.g. means) or other basic estimates (e.g. regression coefficient) AND variation (e.g. standard deviation) or associated estimates of uncertainty (e.g. confidence intervals) |
| ☐ | ☒ | For null hypothesis testing, the test statistic (e.g. *F*, *t*, *r*) with confidence intervals, effect sizes, degrees of freedom and *P* value noted<br>*Give P values as exact values whenever suitable.* |
| ☒ | ☐ | For Bayesian analysis, information on the choice of priors and Markov chain Monte Carlo settings |
| ☒ | ☐ | For hierarchical and complex designs, identification of the appropriate level for tests and full reporting of outcomes |
| ☒ | ☐ | Estimates of effect sizes (e.g. Cohen's *d*, Pearson's *r*), indicating how they were calculated |

*Our web collection on statistics for biologists contains articles on many of the points above.*

## Software and code

Policy information about availability of computer code

| | |
|---|---|
| Data collection | All ionic current recordings were carried out using a high-bandwidth amplifier VC100 (Chimera Instruments). The current data were recorded at a sampling rate of 1 MHz and filtered at 100 kHz. The electrical data were collected using a Matlab based GUI software from Chimera Instruments. |
| Data analysis | All ionic current recordings were carried out using a high-bandwidth amplifier VC100 (Chimera Instruments). The current data were recorded at a sampling rate of 1 MHz and filtered at 100 kHz. A custom-written application in MATLAB (R2022a), the Nanopore App, was used to analyse the translocation events (see supporting information for further details). Briefly, (1) current-time data was loaded and opened using the Nanopore App. (2) The trace was filtered using a 100 kHz low-pass filter and resampled at 1 MHz. (3) The current baseline was tracked and subtracted. (4) A Poisson distribution was used to determine the open-pore current and thresholds. Typically, a threshold of 7 standard deviations above the mean open-pore current was used to isolate identified events. (5) Events above the threshold were classified as relevant events. (6) Event parameters were saved and exported. (7) After isolating individual events, CUSUM (cumulative sums algorithm)76, 77 was used to fit individual peaks to determine secondary peak amplitude, dwell time, and fractional position. A detailed step-by -step data analysis including justification for fitting parameters and thresholds for each analyte type, can be found in the Supplementary Information. All reported errors in the manuscript represent one standard deviation. Statistical analyses were carried out using Matlab (R2022a), Excel (Microsoft Office 365) and OriginLab (2023). The figures were plotted by OriginLab and further imported to Adobe Illustrator (Adobe CC). |

For manuscripts utilizing custom algorithms or software that are central to the research but not yet described in published literature, software must be made available to editors and reviewers. We strongly encourage code deposition in a community repository (e.g. GitHub). See the Nature Portfolio guidelines for submitting code & software for further information.

# Data

Policy information about availability of data

All manuscripts must include a data availability statement. This statement should provide the following information, where applicable:

- Accession codes, unique identifiers, or web links for publicly available datasets
- A description of any restrictions on data availability
- For clinical datasets or third party data, please ensure that the statement adheres to our policy

The main data supporting this study's results are available within the paper and its Supplementary Information. Source data are provided with this paper. The example raw traces in this study have been deposited in the Zenodo database at https://zenodo.org/record/8143395. Additional relevant information is available from the corresponding author upon request.

# Research involving human participants, their data, or biological material

Policy information about studies with human participants or human data. See also policy information about sex, gender (identity/presentation), and sexual orientation and race, ethnicity and racism.

| | |
|---|---|
| Reporting on sex and gender | Age, gender of participants are random. The age and gender are not relevant to this study. |
| Reporting on race, ethnicity, or other socially relevant groupings | Race, ethnicity, or other socially relevant groupings of participants are random and they are not relevant to this study. |
| Population characteristics | The viral samples from patients with different variants who attended Imperial College (London, UK) testing scheme was collected between 2020-2022. The patients samples, from people with different gender and age, were fully anonymised redundant samples (i.e., samples left over after testing). |
| Recruitment | The viral samples were provided from the Imperial College London testing scheme and were from fully anonymised, redundant samples (i.e., samples left over after testing) and retained for assay development and quality assurance and assay validation. The sequencing was part of the college's response to ensure that new variants were detected and to detect otherwise unexplained clusters. The consent to providing the sample and to the testing of the sample was provided at test booking through an online process. |
| Ethics oversight | There was no ethics process needed for using the viruses obtained from the samples after the testing was complete. |

Note that full information on the approval of the study protocol must also be provided in the manuscript.

# Field-specific reporting

Please select the one below that is the best fit for your research. If you are not sure, read the appropriate sections before making your selection.

☒ Life sciences ☐ Behavioural & social sciences ☐ Ecological, evolutionary & environmental sciences

For a reference copy of the document with all sections, see nature.com/documents/nr-reporting-summary-flat.pdf

# Life sciences study design

All studies must disclose on these points even when the disclosure is negative.

| | |
|---|---|
| Sample size | The clinical sample analysis, at least 5 patient samples were tested for each healthy and disease (with different COVID variants) stage. No statistical methods were used to determine sample size. The sample size were decided according to a previous study using the same cohorts of samples. The selection of the sample size took into account both statistical significance and the timeliness of validating experimental performance in exploratory experiments. |
| Data exclusions | No data were excluded. |
| Replication | Values described in the biological samples were obtained from 3 technical replicates. All attempts of replication were successful. |
| Randomization | Patient samples were allocated into different cohorts (healthy, COVID wild-type, delta, omicron) according to the RT-qPCR results before testing. |
| Blinding | Data collection and analysis were not blinded. Blinding was not relevant because the the study is a pilot investigation. The main purpose of the study was to test the feasibility of combining the nanopore sensing strategy and molecular probe system to achieve multiplex sensing for infectious disease which caused by virus. Blinding might complicate the experimental setup or workflow, potentially leading to inefficiencies in this pilot experiment. |

# Reporting for specific materials, systems and methods

We require information from authors about some types of materials, experimental systems and methods used in many studies. Here, indicate whether each material, system or method listed is relevant to your study. If you are not sure if a list item applies to your research, read the appropriate section before selecting a response.

## Materials & experimental systems

| n/a | Involved in the study |
|---|---|
| ☒ | ☐ Antibodies |
| ☒ | ☐ Eukaryotic cell lines |
| ☒ | ☐ Palaeontology and archaeology |
| ☒ | ☐ Animals and other organisms |
| ☒ | ☐ Clinical data |
| ☒ | ☐ Dual use research of concern |
| ☒ | ☐ Plants |

## Methods

| n/a | Involved in the study |
|---|---|
| ☒ | ☐ ChIP-seq |
| ☒ | ☐ Flow cytometry |
| ☒ | ☐ MRI-based neuroimaging |
