# Supplementary figures and images for: Multiplexed detection of viral antigen and RNA using nanopore sensing and encoded molecular probes
Source: Nat Commun. 2023 Nov 14;14:7362. doi: 10.1038/s41467-023-43004-9 (PMC10646045; doi:10.1038/s41467-023-43004-9)

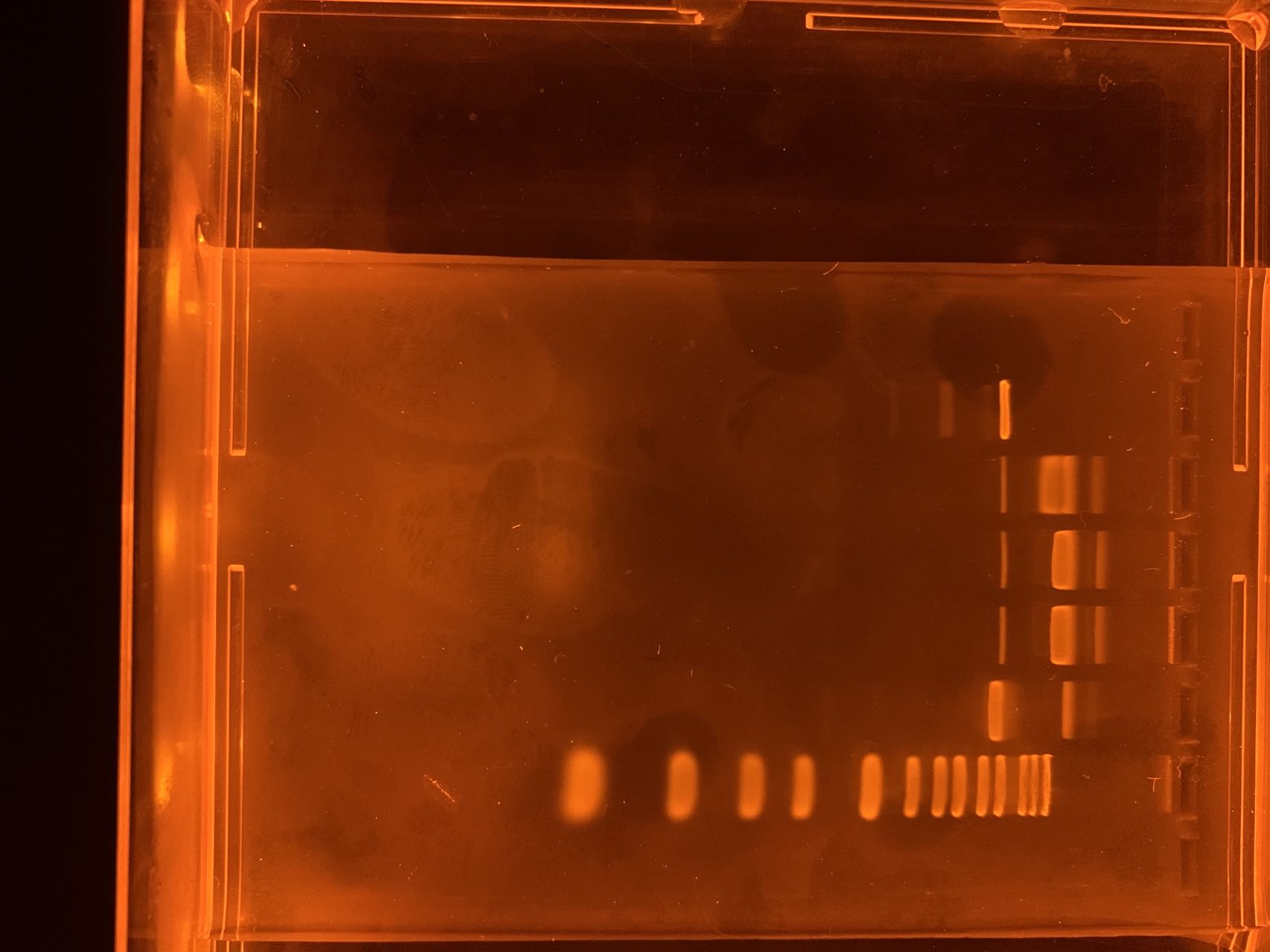

Supplement: Supplementary file 4 — Source data [file 41467_2023_43004_MOESM4_ESM.zip › Source data/SI Fig.12 gel.jpg]

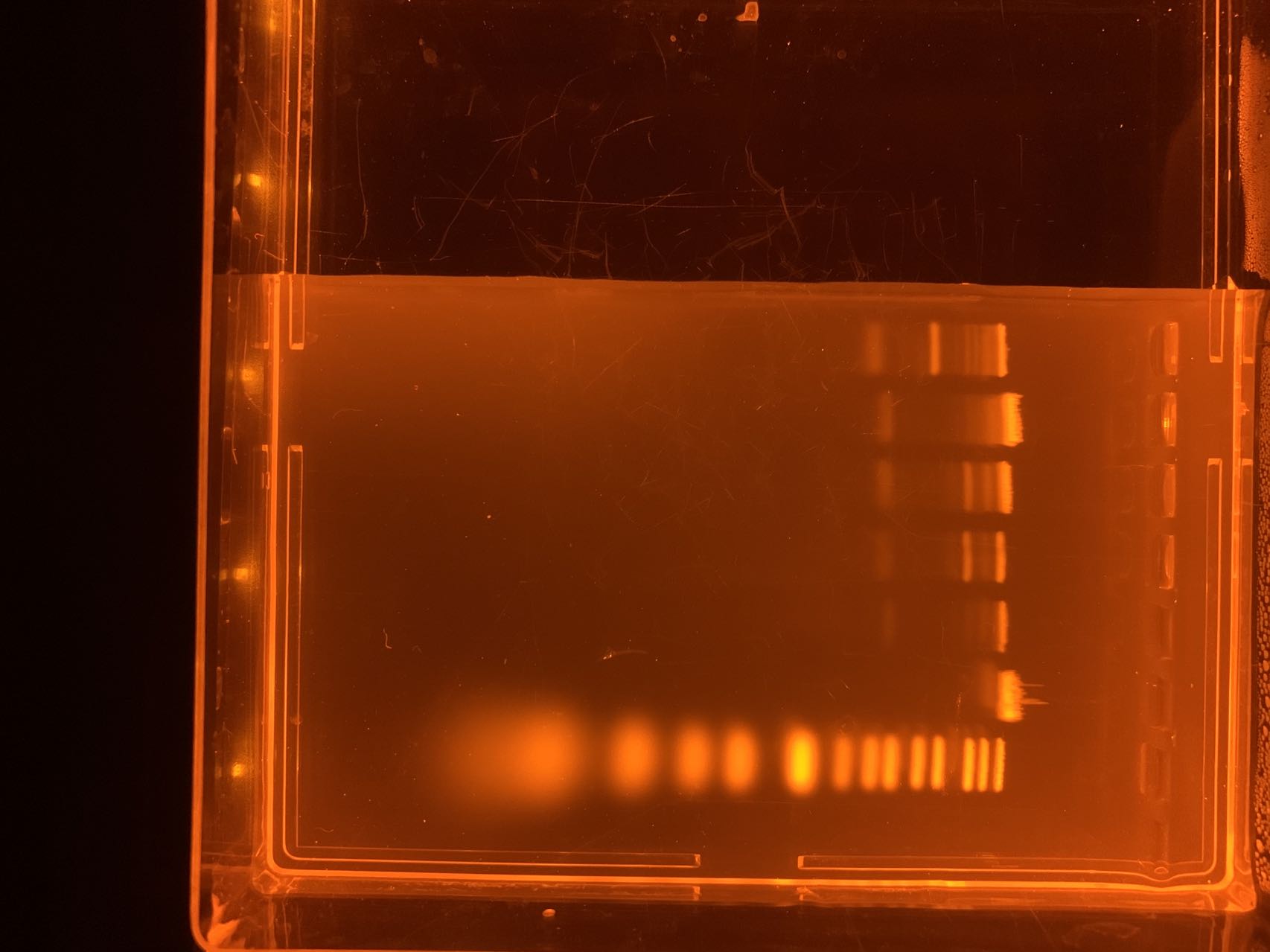

Supplement: Supplementary file 4 — Source data [file 41467_2023_43004_MOESM4_ESM.zip › Source data/SI Fig.19 gel.jpg]

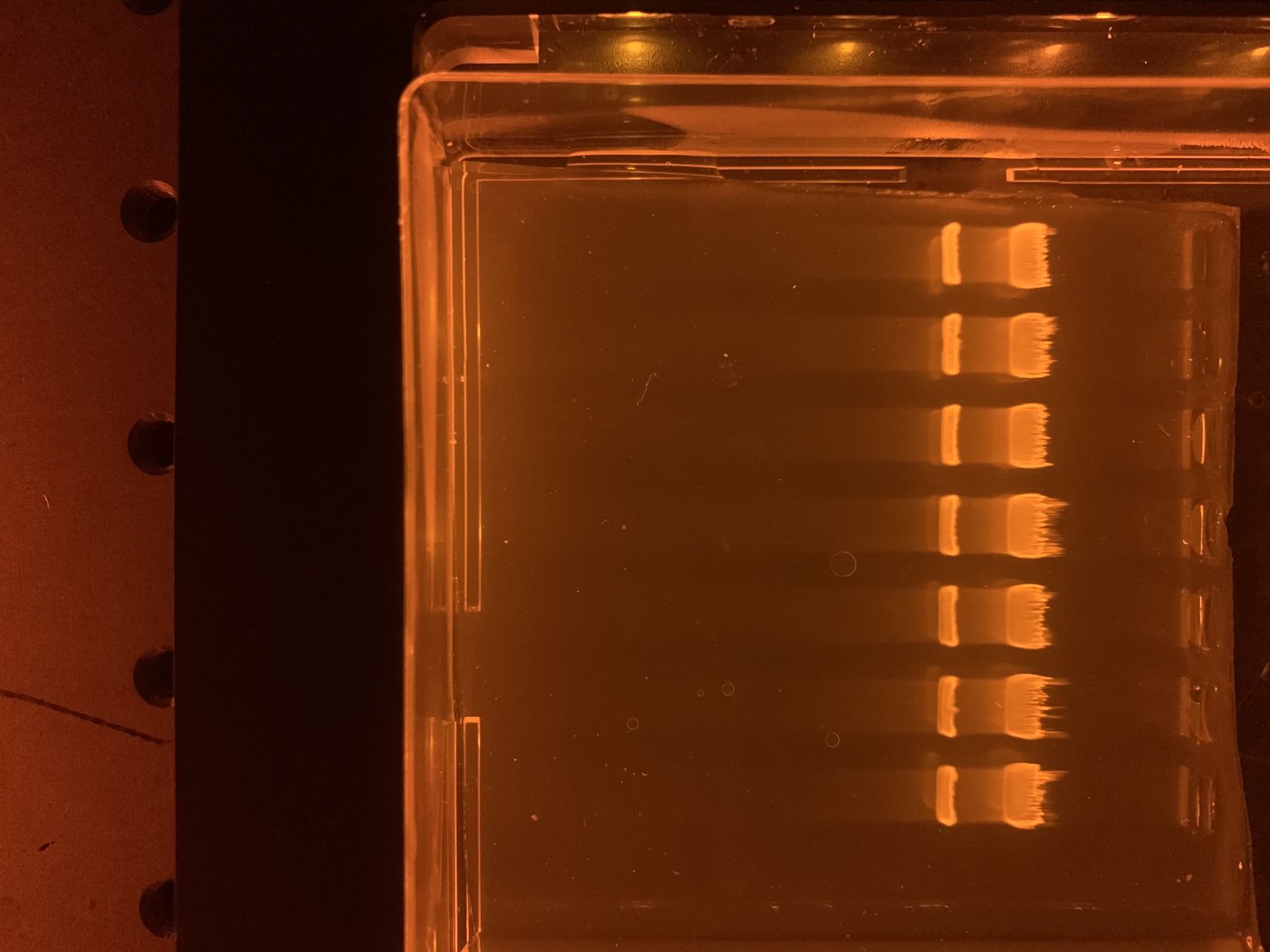

Supplement: Supplementary file 4 — Source data [file 41467_2023_43004_MOESM4_ESM.zip › Source data/SI Fig.2 gel.jpg]
